# A Comprehensive Review on the Optical Micro-Electromechanical Sensors for the Biomedical Application

**DOI:** 10.3389/fpubh.2021.759032

**Published:** 2021-12-02

**Authors:** Anup M. Upadhyaya, Mohammad Kamrul Hasan, S. Abdel-Khalek, Rosilah Hassan, Maneesh C. Srivastava, Preeta Sharan, Shayla Islam, Asma Mohammed Elbashir Saad, Nguyen Vo

**Affiliations:** ^1^Department of Mechanical Engineering, Amity School of Engineering and Technology (ASET), Amity University, Noida, Lucknow, India; ^2^Department of Mechanical Engineering, The Oxford College of Engineering, Bangalore, India; ^3^Department of Electronics and Communication Engineering, The Oxford College of Engineering, Bangalore, India; ^4^Network and Communication Technology Lab, Center for Cyber Security, Faculty of Information Science and Technology, The National University of Malaysia (UKM), Bangi, Malaysia; ^5^Department of Mathematics and Statistics, College of Science, Taif University, Taif, Saudi Arabia; ^6^Institute of Computer Science and Digital Innovation, University College Sedaya International (UCSI) University, Kuala Lumpur, Malaysia; ^7^Department of Physics College of Science and Humanities in AL-Kharj, Prince Sattam Bin Abdulaziz University, AL-Kharj, Saudi Arabia; ^8^Department of Information Technology, Victorian Institute of Technology, Melbourne, VIC, Australia

**Keywords:** optical MEMS (optical micro electro mechanical system), microcantilever, fibre bragg grating, pressure sensor, intraocular pressure, blood pressure, urodynamic, orthodontic

## Abstract

This study presented an overview of current developments in optical micro-electromechanical systems in biomedical applications. Optical micro-electromechanical system (MEMS) is a particular class of MEMS technology. It combines micro-optics, mechanical elements, and electronics, called the micro-opto electromechanical system (MOEMS). Optical MEMS comprises sensing and influencing optical signals on micron-level by incorporating mechanical, electrical, and optical systems. Optical MEMS devices are widely used in inertial navigation, accelerometers, gyroscope application, and many industrial and biomedical applications. Due to its miniaturised size, insensitivity to electromagnetic interference, affordability, and lightweight characteristic, it can be easily integrated into the human body with a suitable design. This study presented a comprehensive review of 140 research articles published on photonic MEMS in biomedical applications that used the qualitative method to find the recent advancement, challenges, and issues. The paper also identified the critical success factors applied to design the optimum photonic MEMS devices in biomedical applications. With the systematic literature review approach, the results showed that the key design factors could significantly impact design, application, and future scope of work. The literature of this paper suggested that due to the flexibility, accuracy, design factors efficiency of the Fibre Bragg Grating (FBG) sensors, the demand has been increasing for various photonic devices. Except for FBG sensing devices, other sensing systems such as optical ring resonator, Mach-Zehnder interferometer (MZI), and photonic crystals are used, which still show experimental stages in the application of biosensing. Due to the requirement of sophisticated fabrication facilities and integrated systems, it is a tough choice to consider the other photonic system. Miniaturisation of complete FBG device for biomedical applications is the future scope of work. Even though there is a lot of experimental work considered with an FBG sensing system, commercialisation of the final FBG device for a specific application has not been seen noticeable progress in the past.

## Introduction

**Figure 1:**
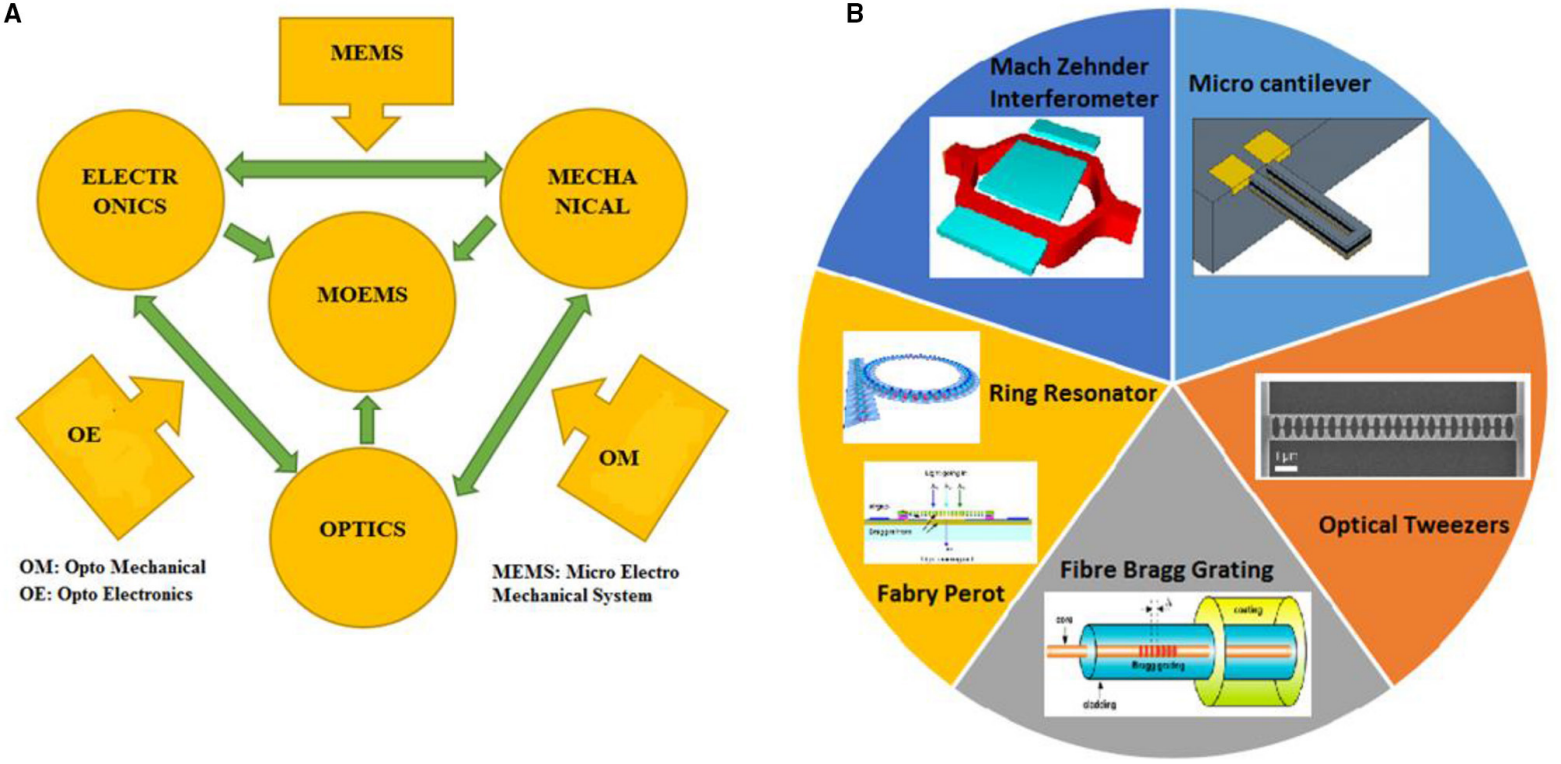
**(A)** MOEMS overview chart, **(B)** different types of Optical MEMS.

Micro-opto electromechanical system or optical micro-electromechanical system combines optical components with micro electromechanical system technology. The micro-level mechanical elements with micro-level motion manipulate the optical signals by actuation provided by electronic structures in a system called micro-opto electromechanical system (MOEMS). Many people are working on optical micro-electromechanical system (MEMS) technology to develop sensitive sensors. Electronic devices size varies from millimetre, micrometre, to nanometre, but mechanical element size ranges from four to five times the electronic devices. This requires sophisticated fabrication technology for optical MEMS. To fabricate the optical MEMS sensor, the technology involved are bulk micromachining, deep x-ray lithography, and surface micromachining. Microfabrication consists of, the process of generating micro ma-chines. Functionality testing of optical and electronic structures is the first phase of MOEMS device fabrication. In this context, the development and analysis of a mechanical element is the second phase of MOEMS device advancement. The third phase involves material deposition, etching, and pattern. The optical design process consists of choosing suitable geometry and assessing optical. Electromechanical design involves various mechanical factors such as stiffness, material parameter, electrostatic, and thermal. MOEMS device design depends on different parameters. Different factors that need to be considered during the design of optical MEMS are mechanical, electrical, thermal, ecological, biological, optical, and chemical aspects based on different applications. The shape and dimension considered during the design of optical MEMS play a significant role in the proper working of optical MEMS devices. Stress distribution due to different geometrical aspects and optimization is a factor that influences the MOEMS design. Figure 1A shows the MOEMS overview chart (1).

Strength, stiffness, hardness, wear resistance, abrasion, creep, and fatigue resistance of the material used during the design are parameters evident in the sound optical MEMS system. The refractive index, an optical property, is the governing factor of any MOEMS device. In the optical design, we know that if the refractive index changes directly affect the performance of any optical MEMS structures, then the particular assignment of refractive index is a must before analysis. The optical MEMS sensor consists of two main parts: (1) the micro sensing element and transduction unit, which powers the supply unit with optical media, and (2) the micro sensing element, which senses the input signals and converts them into output in the required form of the transduction unit. Different types of optical MEMS sensors that are possible in a real scenario are displacement, temperature, bio-sensor, chemical, optical, and pressure sensors. The optical MEMS system can also act as a microactuator by actuating micro actuating elements (2). Figure 1B shows the different types of optical MEMS systems.

This analysis aimed to research the literature on photonic MEMS in biomedical systems. We investigated different studies providing different backgrounds and their relationships. The following research questions (RQ) are specified. RQ1: “What are the major issues regarding using photonic MEMS?” RQ2: “What key success factors are applied to methods that influence performance enhancement?” RQ3: “What are the research gap and scope to design the optimum photonic MEMS devices in the biomedical application?”

## Materials and Methods

This research relied on a systematic review of the MOEMS and its application in the biomedical field. Parallel understanding of available research associated with optical MEMS, such as the basic principle of optical MEMS, different design configurations involved, and its future scope of improvements, were discussed. There are three key phases in this review paper. First, the principle of MOEMS and the different types of optical MEMS. Second, the specific application of optical MEMS. Third, the challenges towards optical MEMS with the current market trend and future scopes. Research papers associated with the basics of optical biosensors, integrated optical sensors, and optical MEMS for biomedical applications, and articles explaining the current status of optical MEMS sensors in the market were selected to help answer the research questions.

Different methods are employed in the design of optical MEMS. It involves modelling MOEMS as the sensor, modelling MOEMS as the actuator, modelling different substrates, beam propagation techniques, computational opto electro mechanics, and high fidelity modelling of the electromagnetic field.

High fidelity modelling of MEMS based on vertical cavity surface emitting method uses Laguerre Gaussian equation. This involves single, double, and multimode equations in consideration.


$$\begin{array}{l} \text{E}(\text{r},\text{z})={\text{E}}_{\text{o}}\frac{{\text{w}}_{\text{o}}}{\text{w}(\text{z})}\frac{{\text{w}}_{\text{o}}}{\text{w}(\text{z})}\text{exp}(\frac{-{\text{r}}^{2}}{{\text{w}}^{2}(\text{z})})\frac{-{\text{r}}^{2}}{{\text{w}}^{2}(\text{z})})\\ \text{ exp}(-\text{ikz}-\text{ik}\frac{{\text{r}}^{2}}{2\text{R}(\text{z})}\frac{{\text{r}}^{2}}{2\text{R}(\text{z})}+\text{ iζ}(\text{z}) \end{array}$$

Maxwell equations are used at the beginning of every optic problem.


$$\begin{array}{l} \text{i}.\text{e}.,\text{div D}=\rho ,\left(2\right)\text{div B}=0,\left(3\right)\text{curl E}=\frac{\text{d}\text{D}}{\text{d}\text{t}}+\text{J}\frac{\text{d}\text{D}}{\text{d}\text{t}}+\text{J} \end{array}$$

where ρ is charge density, J is current density, E is the electric field, B is the magnetic field, and D and H are field quantities that are proportional to E and B, respectively. The last technique involved considering in the design and modelling of optical MEMS is the beam propagation technique. This technique uses Helmholtz time-dependent diffusion equation.


$$\begin{array}{l} \left(-\nabla^{2}-\frac{\omega^{2}}{{\text{v}}^{2}}\frac{\omega^{2}}{{\text{v}}^{2}}\right)\text{ q}\left(\text{x}\right) \end{array}$$

**Figure 2:**
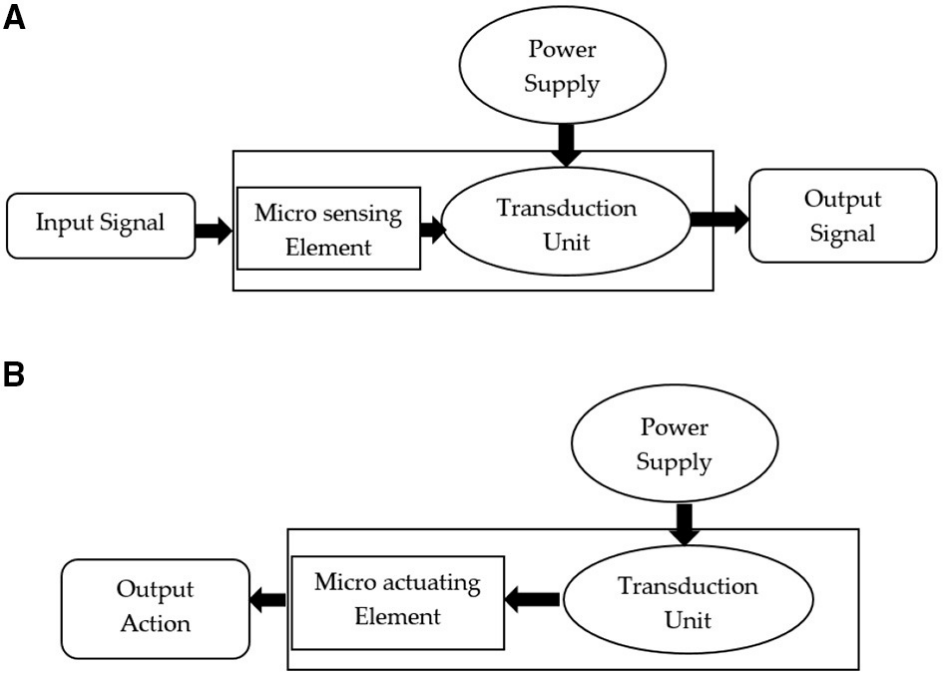
**(A)** MOEMS as sensor, **(B)** MOEMS as actuator.

Many optical MEMS devices incorporate different types of materials for sensing or actuating sensing layers. Different types of micro sensing or actuating elements are thermal forces, electrostatic forces, piezoelectric materials, and shape memory that alloys along with optical MEMS integration in a different environment. Photonic sensing layers silica, optical fibre made up of silicon, are the materials, considered in the optical sensing system. Germanium and gallium arsenide are also used in other conditions like thin films. Along with the other materials, the sensing system also consists of polymer materials. Photonic MEMS as actuator involves movable and fixed sensing elements, cold or hot arm in terms of thermomechanical actuation. Figures 2A,B show different sensing systems with MOEMS.

Major types of optical MEMS such as FBG sensor, optical microcantilever sensor, Mach-Zehnder interferometer (MZI), and photonic MEMS with microfluidic channels, as well as their applications, were discussed here. It was categorised based on its approach to integration with MEMS application and principle of operation, among others. Most of the works of literature in optical MEMS-based microcantilever sensors incorporated with photonic crystal sensing layer, ring resonator structures, and optical readout techniques were discussed. MZI is integrated with the photonic sensing layer or diaphragm for sensing the deflection in the sensing arm. The level of maturity of FBG techniques has reached to commercial stage since they are widely explored in different biomedical applications. Recent advancements in photonic MEMS with microfluidic channels and its design and fabrication were also discussed in this study.

## Type of Optical MEMS Sensors With Application

There are various types of optical MEMS sensors. Depending on the application, different sensing mechanisms had been reported and presented in this manuscript. This section explained the basic principle of all possible optical sensing schemes used in optical MEMS.

### Types of Optical MEMS Sensors

#### Optical MEMS Based Mach-Zehnder Interferometer

Photonic devices generally have extreme sensitivity to the environment and manufacturing, having wide applications. It is a device used to measure the change in phase shift between two beams. In this context, MZI with high sensitivity has three core fibres proposed by Ding et.al. The MZI is considered an optical MEMS sensor that was bringing properties such as elongation or shortening of the core made possible by applying force. Overall bending sensitivity of 15.35 and 3.11 nm/m was obtained (3). MZI is also used to propose the potential measurement of the trajectories of joint angle. Two core trapezoid cone structures have been used for the simultaneous measurement of pressure and temperature. Developed high sensitive MZI for this purpose was sensitive to laboratory seismic noise. A possible micromechanical structure with electro-optical function has been investigated with an In-P material with an integrated optical stress sensor to reduce the seismic noise (4). In another work, Jindal and Raghuwanshi discussed the photoelastic effect induced phase shift during the transmission of light in MZI. Due to maximum pressure at the centre of the diaphragm, the centre portion attached to the MZI gets deflected and brings out the shift in phase and intensity variations. Theoretical analysis of micromachined differential pressure sensor clamped with circular diaphragm and FBG sensor embedded in MZI has been analysed, wherein the sensitivity of this pressure sensor was about 0.2 pm/pa (5).

**Figure 3:**
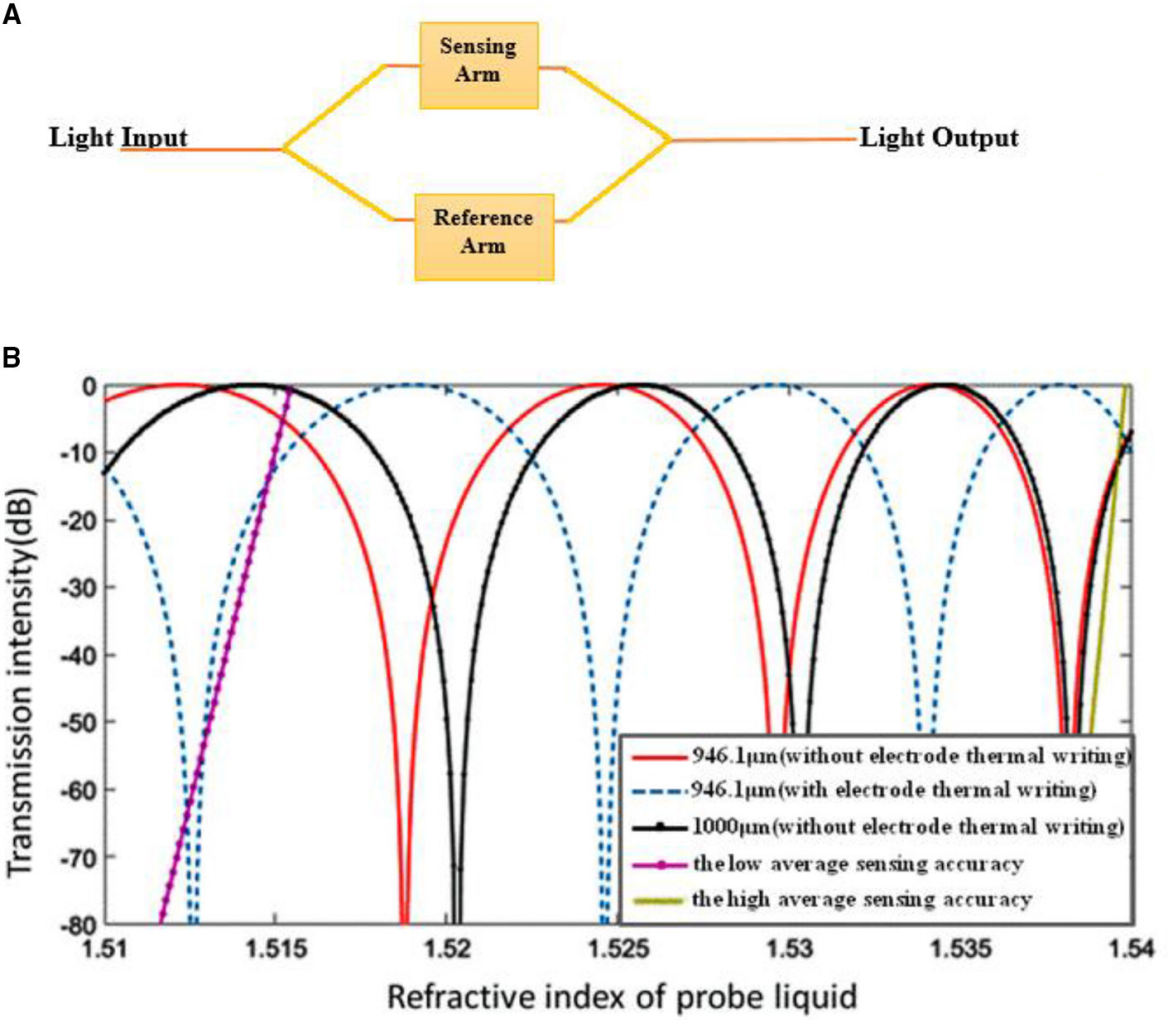
**(A)** Mach-Zehnder Interferometer working process, **(B)** intensity of sensor with various length and refractive index of core (6).

The Mach-Zehnder Interferometer shown in Figure 3A consists of a light input and output and a sensing and reference arm. Left Y-junction which have light input splits the light beam called as a beam splitter and right junction combines the light and called as beam combiner. Two straight waveguides between two Y-junctions locate the sensing arm at one end and the reference arm at the other ends. Output unit, the right end of Y-junction, gives the phase shift, i.e., ΔΦ. Polarised light from laser source passed to two arms of MZI, through input waveguide. Transmission of light through the sensing arm reacts with a biochemical molecule and the overall refractive index changes with the refractive index of the reference arm. Intensity modulation is obtained in the output due to variation in the overall refractive index of a sample in the sensing arm relating to a reference arm with a normal refractive index of the medium. If ΔΦ is the shift in phase, λ is a wavelength, and d is the length of the sensing arm, the effective refractive index is denoted by n_eff_ (7). Equations 1, 2 indicates that MZI sensor output intensity is equal to the oscillating function of interferometer phase change difference.


(1) $$\begin{array}{l} \text{Iout}={\text{I}}_{\text{sen}}+{\text{I}}_{\text{ref}}+2\surd {\text{I}}_{\text{sen}}{\text{I}}_{\text{ref}}\left(\Delta \varphi +\Delta \varphi 0\right) \end{array}$$

where I_sen_ and I_ref_ are the intensity of light passing through the sensing arm and reference arm.


(2) $$\begin{array}{l} \Delta \Phi =2\pi /\lambda \Delta {\text{n}}_{\text{eff}}\text{sensing arm} \end{array}$$

The sensitivity of the MZI is denoted by ΔO/Δn, where ΔO is output power change and Δn changes in refractive index. The phase shift is obtained by Equation 2. In another work, PB Patel and co-authors evaluated a novel optical MZI (OMZI) with rib waveguides. The complete structure has been designed with a silicon rib waveguide. The rib width of the OMZI structure was optimised, and the effect of the rib width on wave propagation was monitored. Change in resonant peak wavelength for refractive index variation due to rib width optimizations was also analysed. It was observed from the results that mono mode behaviour was developed with the rib width of 4 μm and height of 5 μm. The rib waveguide was analysed for wavelength variation of 630 to 1,310 nm (8). In this context, Lin considered a novel electrode temperature writing waveguide based on changing refractive index for the applied heat. Different mode conditions were considered in this sensor design (6). Transmission intensity for the various refractive index of probe liquid is presented in Figure 3B.

**Figure 4:**
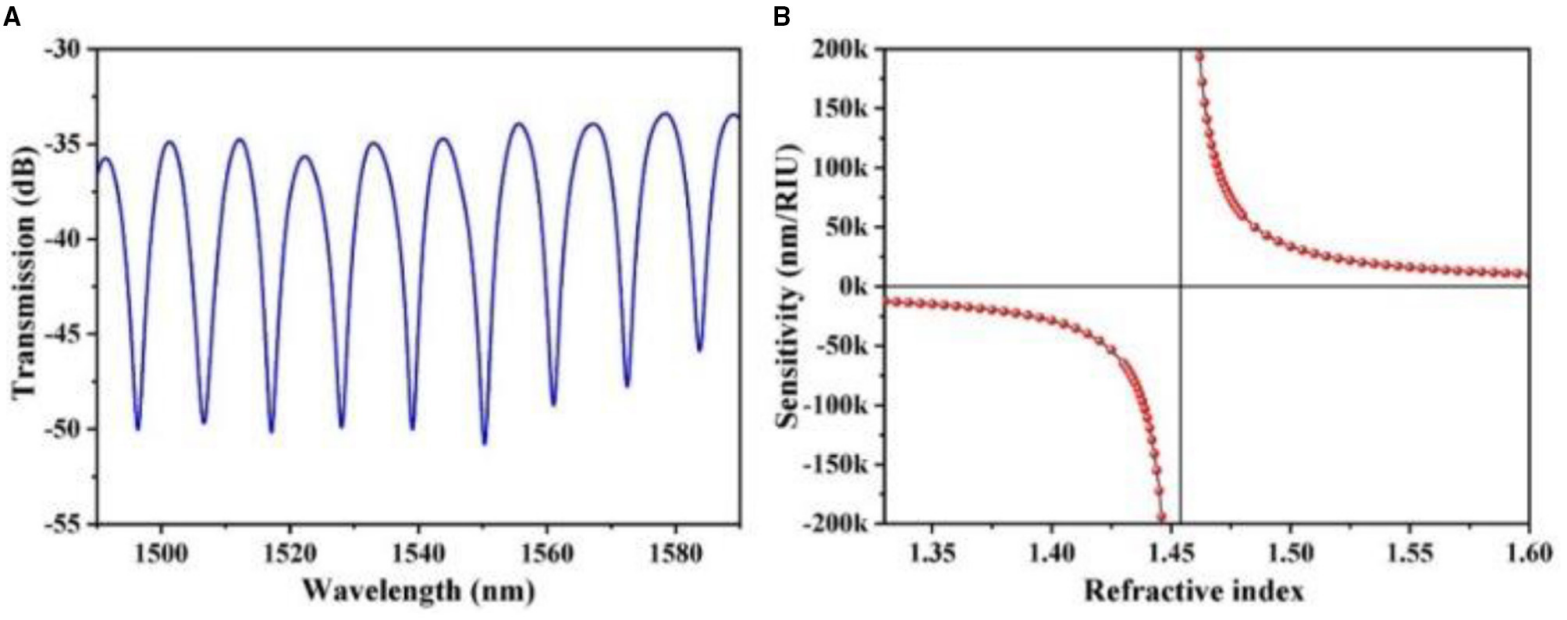
**(A,B)** Transmission spectra and sensitivity calculation of Machzehnder Interferometer (12).

In another work, Yadav et al. discussed optical waveguide-based biosensors on the subwavelength grating which were investigated with a single TE mode in 1,400 to 1,500 nm. The interferometer bend radius of 50 μm was maintained. Two-dimensional FEM simulation was performed with the Comsol Multiphysics software tool. The reference arm was considered with silica material and sensing arm incorporated with air material. There were 2-fold increases in sensitivity after including the sub-wavelength range (9) compared with the MZI proposed with single-mode fibre. Single-mode fibre was coated with methylcellulose. It has shown a humidity sensing range of 45–8% RH. A sensitivity of 0.094% RH was obtained during the simulation. An increase or decrease in the relative humidity has shown the difference of intensities up to 0.03 dB (10). Highly sensitive silicon MZI based ultrasound sensor was discussed by Ouyang et al. In this sensor, one end of the sensing arm was integrated with a thin membrane and another end of the arm was used as a reference. The ultrasound wave was excited on the sensing layer of the interferometer. The low detection limit of 0.38m Pa/Hz was obtained. In preliminary experimental conditions, the image wire phantom was formed. The properties and sensing characteristics of the sensor showed promising application such as ultrasound imaging (11). In this context, high temperature-sensitive MZI was designed with a system of optical fibre offset splicing techniques. The designed sensor has shown sensitivities for external refractive index up to −17,335 nm/RIU and temperature for 32 pm/C. The sensor was constructed with a quartz capillary sandwiched process. The sensitivity of the sensor achieved was 21.2 nm/RIU. The authors concluded its application in many biomedical and industrial concepts (12). It is observed from Figure 4 that sensitivity changing for change in the refractive index.

In another work, Luan et al. discussed a highly sensitive lab on-chip optical ring resonator. Different types of optical ring resonator structures were considered, such as doped silicon-based dual ring resonator system and phase-shifted FBG-based interferometry system. Sensitivity was compared with different configurations (13). In this context, Wang et al. developed an MZI-based sensor for continuous respiratory monitoring. During the development of the MZI-based respiratory monitoring system, it was adequately scrutinised for its respiration rate, tidal volume, and minute ventilation. The designed sensor was constructed with a flexible arch-type structure feature with curvature sensitivity of 8.53 dB/m (14, 15). The study of Zunic et al. designed a MOEMS ultrasonic sensor for photoacoustic imaging. MOEMS sensor was designed and developed with the integration of the MZI. The model was designed and simulated in Comsol Multiphysics. Analysis has been conducted for different materials assigned to layers. Different materials assigned were Si, SiO2, Si3N4, and SMMA. The sensor was designed to operate at 1 MHz (16). The MZI was designed for measuring glucose in serum. The light that was propagating through the sensing system which detected the serum sample with a specific concentration of glucose was analysed. The output power obtained was linear until it reached the value of 1.358 (17).

#### Optical MEMS Microcantilever

**Figure 5:**
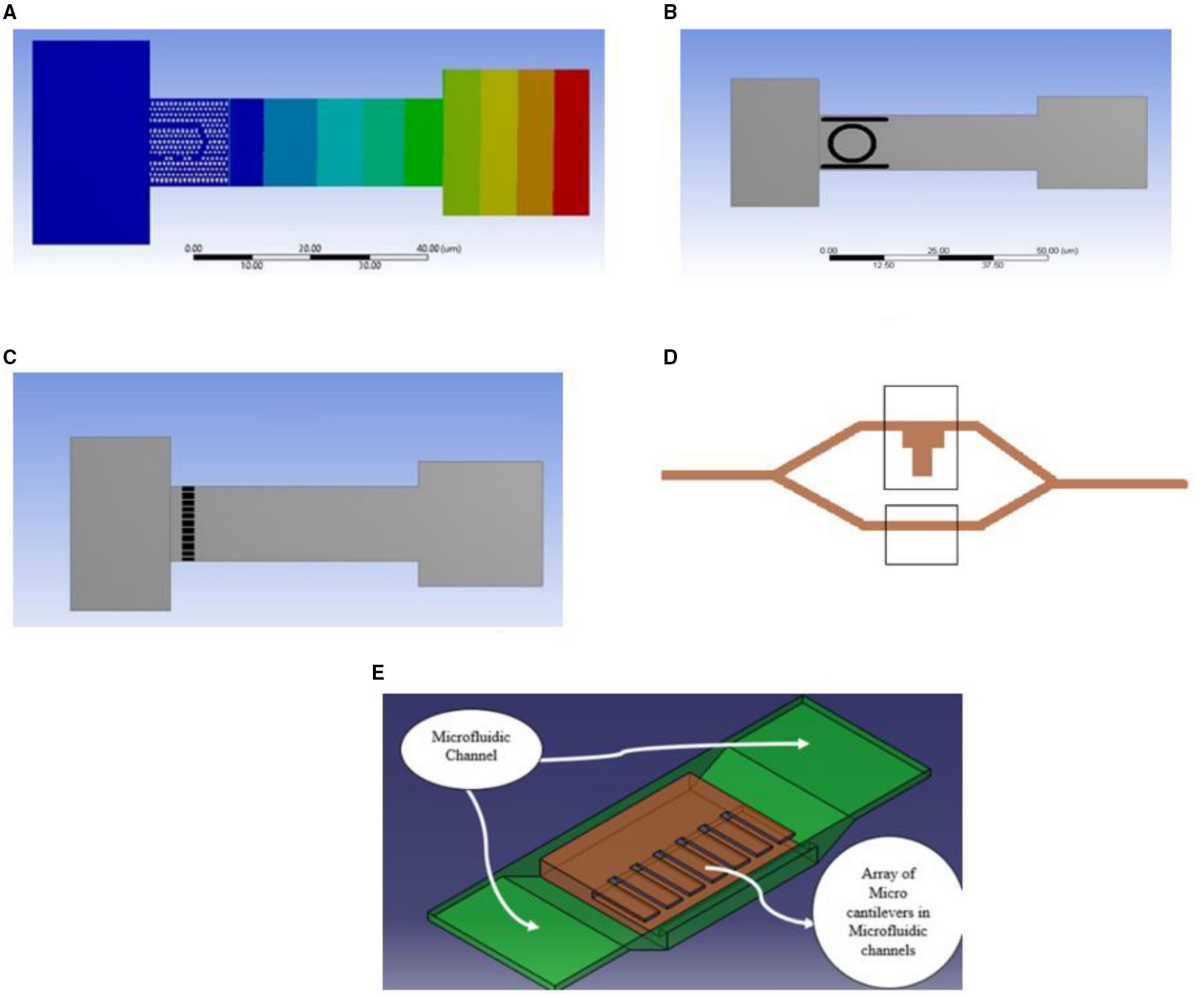
Presents the various Microcantilever: Photonic crystal integrated Microcantilever **(A)**, ring resonator integrated Microcantilever **(B)**, FBG integrated Microcantilever **(C)**, MZI Integrated with Microcantilever **(D)**, array of Microcantilever with the microfluidic channel **(E)**.

Micro-electromechanical system microcantilevers have wide applications in many biomedical aspects, especially most of MEMS biosensor integrated with microcantilever system because of its flexibility in mass sensing, benefits of EMI insensitivity, and micro-sized fewer circuitry parts (18). Several micromechanical structures were configured with the microcantilever sensor which were widely used for label-free biosensor applications. It has been used in many applications and research studies, including the fabrication of monolithic Wheatstone bridge circuits (18, 19) are the analysis of electrical responses of MEMS piezoresistive microcantilevers. Microcantilevers have been widely used in medical applications such as the induced mass change technique for glucose detection (20–22). Microcantilever sensor work is mainly based on maximum surface stress generation due to applied force at the cantilever tip. Combining optical systems with micro cantilever makes the optical sensor system work effectively for change in deflection and maximum surface stress. In terms of label-free sensing, variety of methods can be integrated with microcantilever sensors. They are (1) photonic crystal structure, (2) MZI, (3) optical ring resonator waveguides, (4) FBG, etc. Label-free sensing uses protocols of change in refractive index due to change in surface density instead of a change in mass. In this review, we discussed microcantilevers, which have undergone micromachining technology. Cantilever deflection is highly sensitive to change in maximum stress developed at the surface of the cantilever. Biomolecules, DNA, and Protein molecules can be captured by the targeted surface of the cantilever coated by the adsorption layer of the material, which increases the surface stress. Adsorption in one side of the cantilever brings out differential stress for each deflection of the cantilever. The electrical and optical methods of cantilever readout methods are in commercialisation. Still, the optical system of the cantilever sensor has more advantages than electrical due to its simplicity, lightweight, small size, EMI Insensitivity, less circuitry complexity, reliability, and linear results (23). In this context, Lee et al. discussed a nanomechanical cantilever sensor integrated with a photonic crystal microcavity resonator. Resonant wavelength shift was observed for each increment of applied force. It has shown a promising future of application in the detection of biomolecules (24). In another work, Xiang and Lee proposed optical readout chemical sensing based on microcantilever sensors. Photonic crystal microcavity resonators integrated with microcantilevers in a fluidic channel for chemical sensing with water and air were investigated. Microcantilever sensitivity in both air and water for the designed cantilever was analysed, wherein minimum detectability obtained in water was less than in the air (25). Microcantilever was used for the detection of specific biomolecules. Overall geometry was designed, allowing simple microfluidic delivery channels to make adsorb to the surface of microcantilever (26). An optical readout mechanism was used for analysing the optical MEMS sensor. Ptolemy-II is a tool considered for experimental validation of designed optical MEMS sensors. Microcantilever was integrated with many other photonic technologies for the development of optical MEMS-based sensors. Photonic crystal microcavity with micro cantilever is applicable in a different biomechanical application. Optimising photonic crystal structure integrated with micro cantilever cavity for label-free biosensing application has more scope in improving sensitivity. Variation of ring thickness and ring radius is an essential factor affecting the overall sensitivity of the ring resonator integrated with the microcantilever. From **Figure 7A**, we can see the microcantilever array is embedded with microfluidic channels for analysing the chemical solution (30, 31). Figures 5A–E shows different methods of optical system integration with Microcantilever including a microfluidic channel.

In this context, Steven et al. discussed carbon nanotube-based carbon mechanical resonators. It has been used to detect different molecules in the air. The length of the cantilever ranges from 500 μm to 5 mm. The porosity of carbon resonators varied with a density of 102–104 kg/m^−3^. Significant applications in biomedical disease detection and pathogens in air and gas environments have been analysed (32). SU-8 polymer material based microcantilever designed and analysed for disease detection. From the evolution of SU8 Polymer-based surface stress microcantilever. The selection of material, geometrical design parameters, and challenges faced in the current market are discussed (33). By considering different materials, maximum deflection for each specific material of microcantilever is analysed. Microfluidic channels have been designed and analysed for blood flow and viscosity generation for the same sensor configurations. Pressure-induced on microcantilever due to an increase in blood viscosity is investigated. The performance of different microcantilever sensors for different materials is carried out. They concluded that SU8 polymer material gives the best displacement and strain distribution range along the microcantilever (34).

#### Photonic Crystal Pressure Sensor

**Figure 6:**
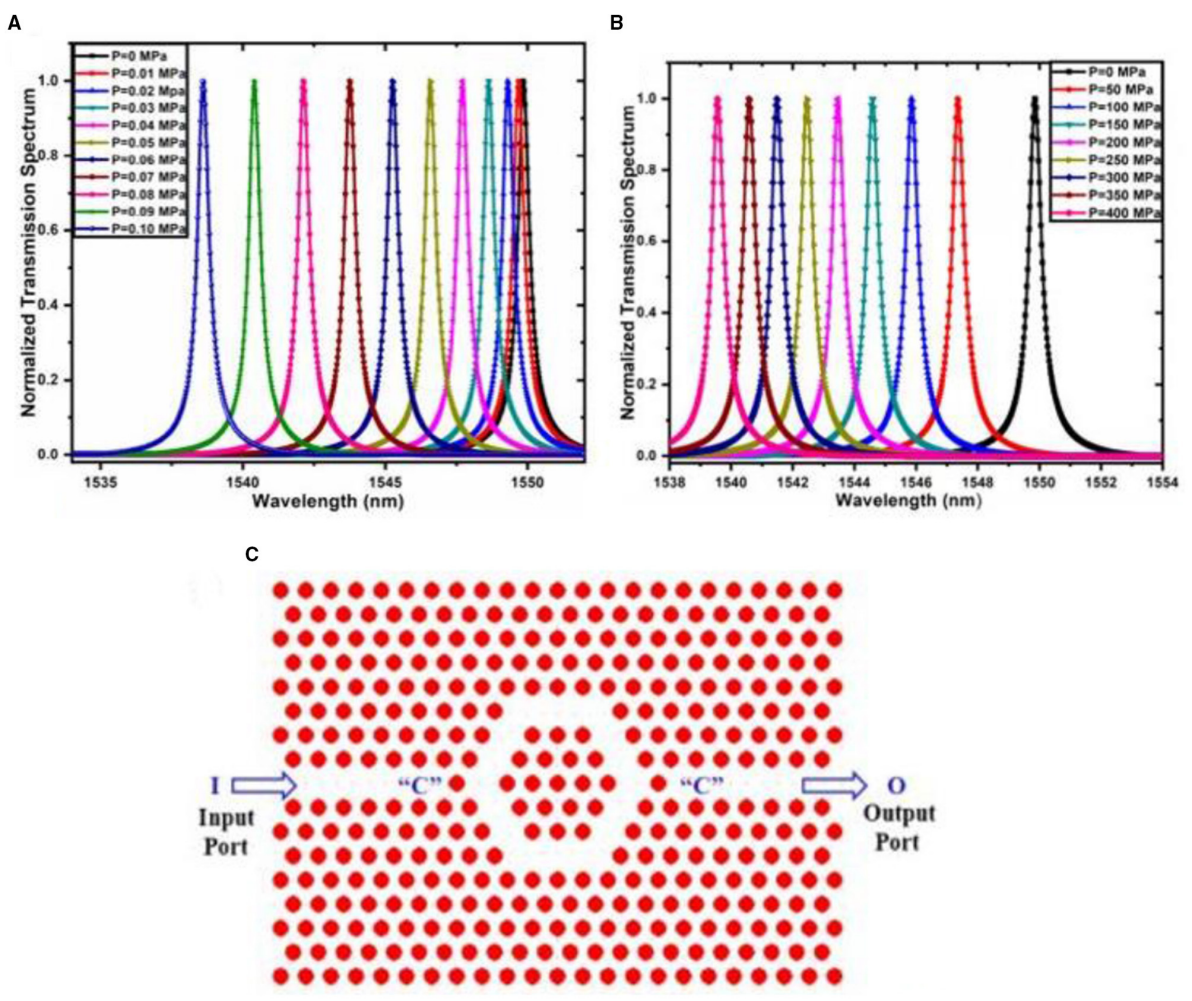
Peak resonant wavelength for sensing structure **(A)** fixed at one end. **(B)** Fixed at both the end (35). **(C)** Hexagonal photonic crystal ring resonator-based pressure-temperature sensor (36).

The photonic crystal is an optical nanostructure that affects the photon motion much similar to the motion of electrons affected by the ionic lattices. Photonic crystal structures are susceptible to change in refractive index by mechanical deformation. A minute change in deflection causes the overall refractive index of the structure to change. Agarwal et al. discussed a photonic crystal-based micro-opto electro mechanical system sensor in this context. Sensor configuration consists of the integration of a photonic crystal sensing layer in a bridge-type silicon structure. In this context, two cases of sensing structures were investigated, i.e., in the first case, the bridge structure was fixed at one end, while in the second case, it was fixed at both the end. The pressure is applied at the centre of the bridge in the second case. In the first case, the pressure was applied at the free end tip of the sensing structure. The pressure is applied from 0 to 400 MPa for both sensing configurations. The analysis observed that the structure fixed at one end shows higher sensitivity of about 68.296 nm/MPa. Structure fixed at both ends shows a sensitivity of 0.134 nm/MPa. The figure shows the resonant peak obtained for the structure fixed at one end. We can see from Figure 6 that there is a distinct shift in wavelength for the different pressure range from 0 to 400 Mpa. The wavelength range obtained for structure with one end fixed is 1,550 to 1,535 nm (35). The peak resonant wavelength for each pressure increment is shown in Figures 6A–C.

In another work, a hybrid dual-core photonic crystal fibre-based pressure sensor was designed. Elliptical and circular shaped air holes were constructed in photonic crystal fibre pressure sensor configuration. Under different applied pressure, the mode behaviour of the sensing configuration was analysed. The sensor was designed for hydro pressure applications. It was found that a sensor length of 6 cm have sensing range from 0 to 1,000 MPa (37). In this context, Rajasekar et al. discussed a hexagonal photonic crystal ring resonator structure for sensing pressure and temperature. The pressure range considered was 0.04–6 GPa and the temperature range obtained was 5–540°C Bandgap analysis of each sensing configuration was carried out to monitor the range of frequency. 2D hexagonal photonic crystal resonator arranged with a circular rod in air configuration was considered during the analysis. In another work, a 2D hexagonal lattice of six hexagonal-shaped rings of hexagonal lattice sensing configuration was micromachined on tip of the microcantilever. The concept of using a microcantilever tip is to get a wide range of pressure. The pressure was applied from 1 to 10 Mpa with a resolution of 10 MPa (36, 38). The hexagonal ring resonator has shown better sensitivity up to 250 nm/MPa. The modified refractive index after applying pressure for 2D photonic crystals was calculated by the following Equation 3 (36).


(3) $$\begin{array}{l} \text{n}=\text{no}-\left({\text{C}}_{1}+{2\text{C}}_{2}\right)\sigma \end{array}$$

where no is the refractive index at zero pressure, C1 and C2 are photoelastic constants and σ is the stress generated at the sensing structure due to pressure. Figure 6B shows the photonic ring resonator structure.

#### Fibre Bragg Grating Sensors

Fibre Bragg Grating has a unique characteristic to perform as a sensing system. The core of the FBG is exposed to intense laser light. Refractive index increases as there is an increase in exposure to the laser light. In this context, Werneck et al. discussed the basic principle of FBG sensors. A periodic change in the refractive index in the core segment of optical fibre was grating fibre. Refractive index variation due to applying pressure or temperature led to pitch change in FBG. The most common choice of fibre optic-based sensor is FBG sensors. The ultrasonic laser inscribing process generated the periodic refractive index on a core segment of optical fibre. An FBG suitable for measuring strain in a product or device has experienced pressures throughout the process. The multiple FBG sensors could be embedded under any structure to get the spectrum shift from the FBG interrogator. The most application of FBG sensors was based on the structural health monitoring system. In structural health monitoring, FBG sensor placement is the most crucial part of the research. Since there is an application of the thermal or structural load on the FBG sensor, installing an FBG sensor inside the structure requires prior knowledge of the structural behaviour. The FBG sensor placement study was conducted in many mechanical applications like the placement of fibre in the tensile test specimen, placement of fibre in wings of the aeroplane for structural health monitoring, or in the case of rail monitoring, during the movement of train wheel for a specific period. From a biomedical point of view, most blood pressure measurements and temperature measurements were in progress with the FBG system. In many applications, the FBG sensor was used as non-invasive. Most FBG Interrogators will provide the raw data in the form of voltage; the same is converted in wavelength for each change in microstrain and feed rate of FBG sensor (27).

The study of Bonefacino et al. discussed ultra-fast polymer optical FBG inscription for medical devices in this context. The fibre consisted of polymethyl methacrylate material. The extraordinary result was obtained during the experimentation. New dopant diphenyl disulfide causing positive refractive index change was utilised during the process. This made sensitivity high when FBG was considered with PMMA to compare with silica fibre. The sensitivity of the FBG sensor obtained was −55 pm°C^−1^ (39). The strain from the FBG sensor was also used to calculate the curvature and torsion of fibres. The catheter was used for reconstruction techniques. The FBG embedded catheter was used as a shape sensor. The maximum reconstruction error found was 1.05 mm. The authors concluded that shape sensing with flexible biomedical instruments is feasible with the FBG sensor (40).

**Figure 7:**
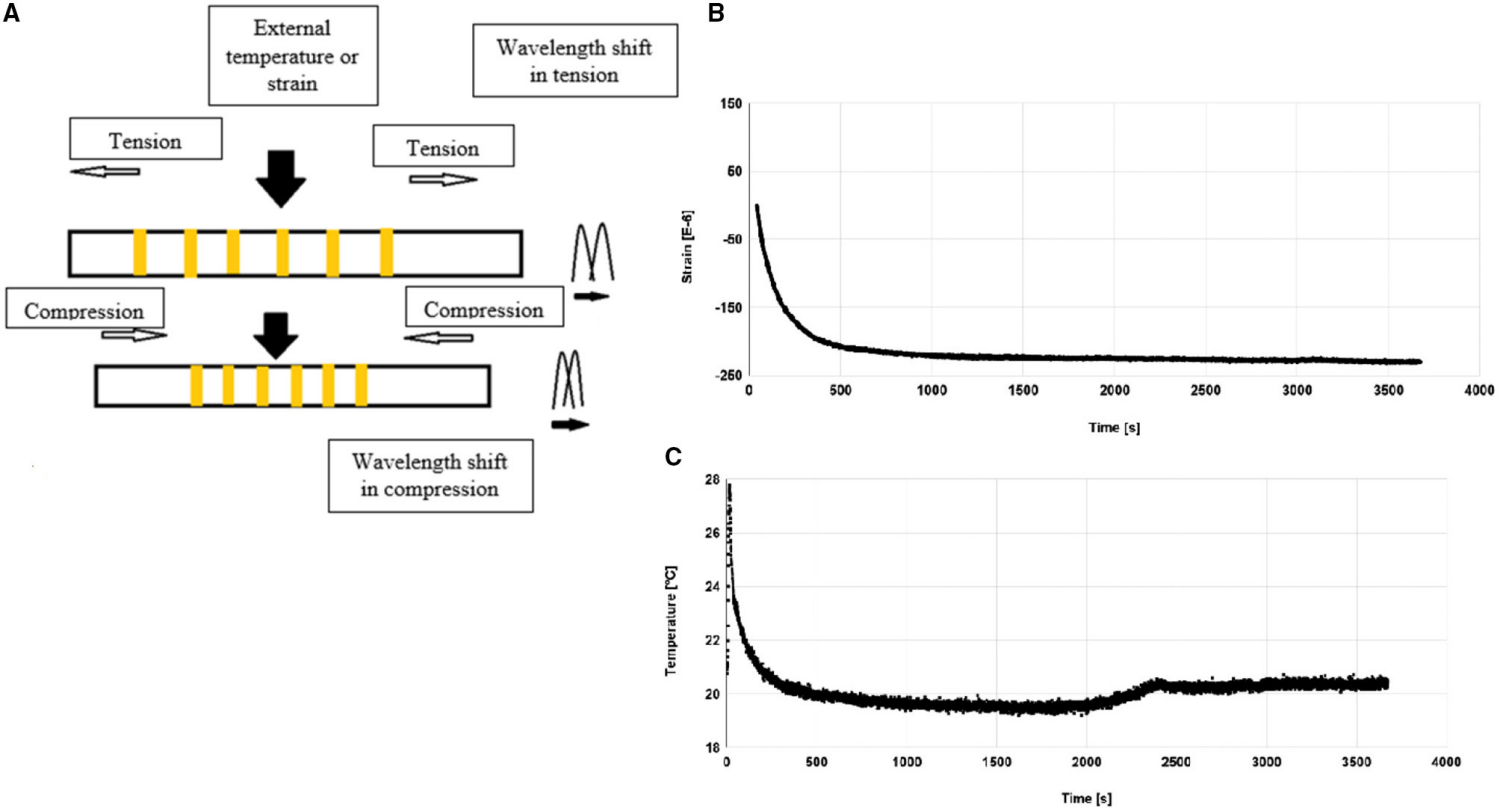
**(A)** Fiber bragg grating working process during the application of pressure (27). **(B)** Strain evolution of bulk flow resin during polymerization (28). **(C)** Temperature evolution of bulk flow resin during polymerization (29).

Along with these applications, the uses of FBG in civil engineering applications, biomedical applications, military, and marine applications were discussed. Depending on the type of application, FBG is designed with long or chirped grating, which is uniform and tilted (40, 41). In this context, Zheng et al. discussed 3D hydrogel actuators driven by light and generated by two-photon polymerization. The photoresist of gel was produced by Fe_3_O_4_. The size of the hydrogel actuator was 26 μm and the response time of the sensor was 0.033 s. The designed actuator has good controllability and repeatability (42). On-chip optomechanical sensing configuration has been established. Sensor configuration consisted of a dual-channel integrated waveguide and directional coupler. The dynamic range of the sensor was 30 nm with displacement imprecision of 45 fm/Hz (43). In another work, Linh et al. proposed an FBG-based optical pressure sensor. The pressure sensing element was constructed with Fabry-Perot based pressure sensor. Fabry-Perot pressure sensor with a different cavity length was optimised. Obtained pressure sensitivity was −0.672 rad/MPa. The proposed sensor was having a very high-temperature sensing ability (44). In another work, optical fibre-based pressure sensing application system was developed for pulse transit time measurement. Pulse transit time measurement system is helpful in cuffless blood pressure measurement. Electrocardiograms and photoplethysmographs had been monitored with a pulse transit time system. Plastic optical fibre was deployed as a transport medium in the chest part of the human to monitor the pulse pressure. Research work carried out here considered pulse transit time (PTT) in three different positions, i.e., finer, wrist, and foot (45). Complementary metal oxide semiconductor (CMOS) MEMS-based membrane nanomechanical sensor is designed and developed for molecule detection. Results shows that small molecules significantly impact the human body, wherein detecting such a molecule is challenging in many sensing systems. This membrane bridge sensor is used to detect phenytoin in different concentrations. The detection limit of this sensor is 4.06 ± 0.15 μg/ml. It is used as a biosensor as well chemical sensor. In this context, Raghuwanshi et al. carried out a sensitivity analysis for TiO_2_-coated FBG sensor for chemical detection. A TiO_2_-based FBG sensor was developed with Cu-Vapour-based laser writing. The thickness of the coating has been optimised to get better sensitivity and FBG design parameters. The sensor was able to detect the minute change in adulteration with accuracy 0.01 ppm (46, 47). In the study of LV, C and co-authors, they discussed invasive FBG based pressure sensors for palpation of tissue surgery. The finite element method has been used to analyze the static and dynamic performance of the sensor configuration. Optimum sensor design output has been given to 2.55 mN. The pressure range considered was 0–5 N (48). In another work, the effectiveness of sensing configuration has been presented by Khan et al. for the pose measurement system of different medical instruments by FBG sensor. The results of the FBG sensor were converted to strain. Strain calculations were made by comparing strain gauge parameters. Mean error in the orientation and position was observed to be <4.69 mm and 6.84°. Different poses of catheters and many other medical instruments by experimental data were obtained (49). In this context, Antunes discussed the mechanical testing of dental resin. In real-time usage of dental resin, resin materials had undergone failure due to the shrinkage polymerization. This caused the breakage of gaps, which interfaced the dental resin with the tooth. A laboratory experiment was conducted to cheque the ability of different resin materials and their failure point by using an FBG sensor. The FBG sensor was placed in the dental model (28). Variation of strain concerning time with FBG sensor is shown in Figure 7B. Figure 7A shows the FBG sensor processing system. Performance comparison of different sensors is shown in Table 1.

**Table 1:** Performance comparison of selected sensors.

**Type**	**Configuration**	**Strategy**	**Mode**	**Detection limit**	**Sensitivity**	**References**
Interferometer	MZI	Three core fibre for measurement of directional bending	TE	n.r.^a^	Bending sensitivity −15.35 nm/m^−1^ and 3.11 nm/m^−1^ Temperature sensitivitiy 0.043 nm/°C and 0.041	(3)
	Diaphragm Integrated MZI	Analysing to light intensity with diaphragm deflection	TE	n.r.^a^	−0.2 pm/Pa	(5)
	MZI	Lab on chip biosensor with MZI Phase modulator		<0.05 L/min), a high resolution (0.005 L/min), and a fast response time (12 millis	n.r.^a^	(6)
	MZI	Methylcellulose coated MZI for humidity sensing	TE	45–85% RH	0.094 dB/%RH	(10)
	MZI	MZI Ultrasound Sensor	TE	0.38 mPa/Hz^1/2^	0.62 mV/Pa	(11)
	MZI	MZI temperature sensor	TE	n.r.^a^	21.2 nm/C	(12)
	MZI	MZI Respiratory monitoring	TE	n.r.^a^	Sensitivity upto 8.53 dB/m-1	(14)
Photonic MEMS /Microcantilever	Photonic Microcantilever	Photonic Microcantilever for chemical analysis	TE	0.6 mum and 0.0098% in water and 0.812 mum and 0.0144% in air,	n.r.^a^	(25)
	Optical MEMS microcantilever	Various shape microcantilever integration with Optical MEMS	NA	81.55 μg−28.39 mg (polyimide material –Triangular cantilever. 50.97 μg−23.996 mg (Parylene material	n.r.^a^	(30)
	MOEMS	MOEMS nanomechanical sensor	TE	0–0.10 MPa;(One end fixed) 0–400 MPa; (both end fixed)	68.296 nm/MPa (fixed one end) 0.134 nm/MPa (Both end fixed)	(35)
	Photonic crystal fibre	Hybrid dual-core photonic crystal fibre	TE	0 to 1,000 Mpa	−11.6 pm/Mpa.	(37)
	Photonic crystal	Pressure and Temperature sensor with hexagonal resonator	TE	0.04 to 6 GPa	n.r.^a^	(36)
	Microcantilever Hexagonal ring resonator	hexagonal ring and micromachined cantilever tips on 2D silicon photonic crystal	TE	1–10 MPa, 10–100 MPa, 1-MPa −20MPa, 20 MPa−10 GPa	n.r.^a^	(38)
Fibre Bragg Grating	FBG sensor for biomedical	Polymer optical fibre Bragg grating analysis	TE	n.r.^a^	54.2 pm %RH−1 ± 0.14%RH	(39)
	FBG for biomedical	Integrated nano-optomechanical displacement sensor		Displacement imprecision of only 45 FM/Hz1/2 as well as a large dynamic range (>30 nm).	n.r.^a^	(43)
	Fibre pressure sensor	Multi-Point Optical Fibre	TE	n.r.^a^	−0.672 rad/MPa	(44)
	CMOS based Nanomechanical sensor	CMOS MEMS-based nanomechanical sensor for molecule detection	NA	4.06 ± 0.15 μg/mL	n.r.^a^	(45)
	FBG force sensor	FBG force sensor for tissue palpation	TE	0–5 N	n.r.^a^	(48)

a *Not reported*.

Figure 7C shows the temperature variation during resin polymerization in dental material. The shift in wavelength was obtained for change in these parameters. Strain dependence of FBG sensor can explain with the following mathematical expression (4) and (5) (29).


(4) $$\begin{array}{l} \Delta \lambda /\lambda \text{\_}0={\text{n}}_{\text{eff}}/\Lambda \text{\_}\left(\text{eff\_}\Lambda \right)=\left(1+\left(1/{\text{n}}_{\text{eff}}\right)\delta {\text{n}}_{\text{eff}}\delta \varepsilon \right)\Delta \varepsilon \\ =\left(1+{\text{p}}_{\text{e}}\right)\Delta \varepsilon \end{array}$$

(5) $$\begin{array}{l} \Delta \lambda \lambda \text{\_}0=k\Delta \varepsilon \end{array}$$

where k = k factor of bragg grating, pe = photo elastic constant = 0.21, and Δλ/Δε = 1.2 pm/(μm/m) - Strain sensitivity for FBG at 1,550 nm

Fibre Bragg Grating as temperature dependent has below expressions;


(6) $$\begin{array}{l} \Delta \lambda /\lambda \text{\_}0\\ =\Delta {\text{n}}_{\text{eff}}/\Lambda \text{\_}\left(\text{eff\_}\Lambda \right)=\left(1/\Lambda \left(\Lambda /\delta \text{T}\right)+\left(1/{\text{n}}_{\text{eff}}\right)\delta {\text{n}}_{\text{eff}}\delta \text{T}\right)\Delta \varepsilon \\ =\left(\alpha +\zeta \right)\Delta \text{T} \end{array}$$

α – coefficient of thermal expansion

ζ- thermo-optic coefficient

α = 0.55 × 10^−6^/°C

ζ = 5.77 × 10^−6^/°C.

### Application of Optical MEMS

**Figure 8:**
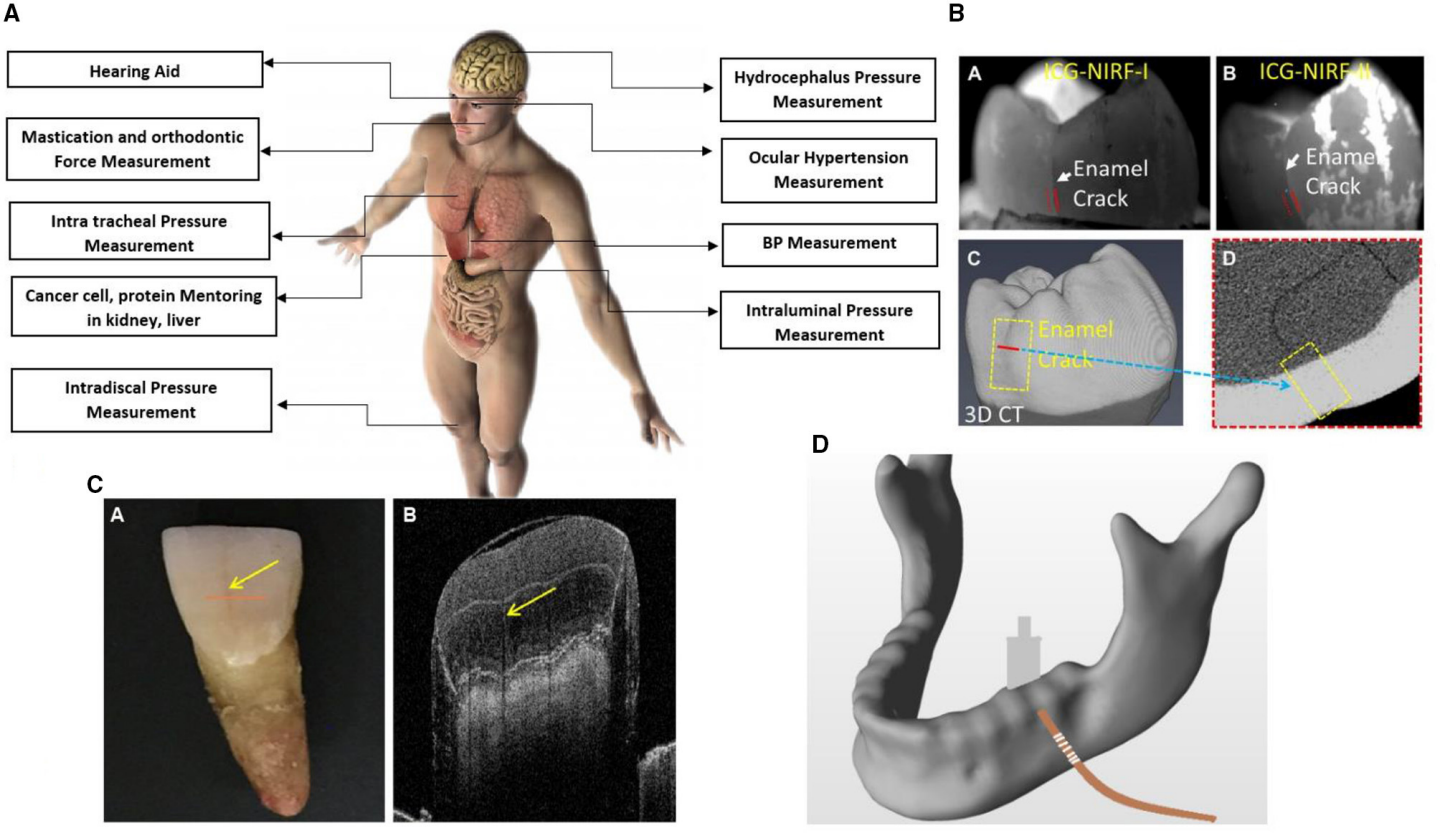
**(A)** Optical MEMS sensors application in the human body (50). **(B)** Dental X-Ray imaging of cracks by green near infrared imaging (51). **(C)** Dental OCT imaging (53). **(D)** FBG integration with human mandibular (52).

Miniaturisation of devices or various micro mechanical elements is a critical concept in developing new optical MEMS technology. Optical MEMS are the microdevices that measure and manipulate various mechanical parameters such as pressure, temperature, shape, stress, strain, etc. Optical MEMS has plenty of applications in present days, e.g., in industry, biomedicine, automobile, and aviation. In this paper, we selected the critical application of optical MEMS in the biomedical field. The primary application discussed here was dental, human spinal disc, optical MEMS in the human respiratory system, the optical fibre in gait motion, blood pressure measurement, and bladder pressure monitoring, among others. Detailed discussion on the advancement of optical MEMS in the stated different applications is presented below. Most of the applications had used optical fibre-based FBG sensing systems (Section Fibre Bragg Grating Sensors) for real-time applications. Microcantilever integrated different types of photonic crystal structures (Section Optical MEMS Microcantilever) were modelled and simulated for various applications. Figures 8A–D shows the Optical MEMS sensors applications in the human body.

#### Dental Application

Incorporating the optical sensor for the dental caries monitoring in the dental application is a new innovative technique. Evaluating the dental filler materials and monitoring the structural behaviour of braces and aligners are essential criteria in dental health monitoring. Recent dental structural behaviour evaluations monitored by the FBG technique were discussed in this review. Evidence suggests that orthodontic tooth movement and biting force can be monitored with photonic MEMS technologies. The work of Silvia et al. proposed a 3D intraoral scanner to map the 3D image in dental restoration accurately. Dental caries is a major problem in dentistry. Global disease study in the year 2016 has been predicted that in the total world of population, wherein 3.5 billion people have been remarked with oral diseases such as tooth decay. Cracked tooth caries are complex tissue lesions that severely affect the quality of life of a person. It has remained a mystery to solve the non-invasive method to detect the cause of caries and crack of the tooth in humans. Nevertheless, early detection of caries is made possible by FBG based optical sensor (53). Figure 7 shows the optical MEMS application overview.

In another work, Pyo et al. discussed the feasibility of using optical coherence tomography in dentistry. Analysis has been carried out by changing the fringe numbers, clarity of dental images has been investigated with optical coherence tomography (OCT). OCT has more advantages for exploring a more complex tooth portion (50). Hybrid technology called photoacoustic imaging high-resolution ultrasound could also project deep tissue with high contrast optical images. Figures 8A,C, show dental scanned images. Strain patterns for different orthodontic devices were evaluated. Regarding this, an orthodontic appliance with a disjunctor bar on the left side and without a disjunctor bar was compared for strain pattern obtained for transferred forces to the dental arch, teeth, or maxilla (54). Figure 8D shows the placement of the FBG sensor human mandibular model. In this context, the FBG sensor was used to measure material expansion because of a dental implant. The effect of thermal strain on FBG sensors has been widely studied. The hardening phase of the material expansion process has been investigated concerning the optical MEMS sensor. Electromagnetic interference (EMI)-insensitive, small dimension bite force device has been developed by FBG technology for measuring. Obtained results showed better resolution compared with the traditional devices available in the market. The optical system was also used in strain measurement of the tibia in bone for decalcification. The different bone sample has experimented for different percentage of decalcification. Since there is a loss in calcium percentage in bone, an increase in strain behaviour was observed. Better resolution over the result was obtained compared with QCT, QUS, and DEXA. These traditional techniques had the drawback of inaccurate values and exposure to radiation (51, 55). In another work, the surface roughness of rot carriers was analysed using optical profilometry. A total of 20 extracted teeth from a patient was examined for root caries, and surface roughness was analysed with profilometry. This clinical study was carried out to protect the non-cavitated surface from cavitation by prior accession of the status of a tooth. In the dental clinical examination, there was no clinical test process to detect hard tissue. This need in the dental field is the reason for the development of optical polarisation imaging systems. This imaging system could assist in dental calculus caries and cracked tooth (52, 56).

#### Strain Monitoring Human Spinal Disc

**Figure 9:**
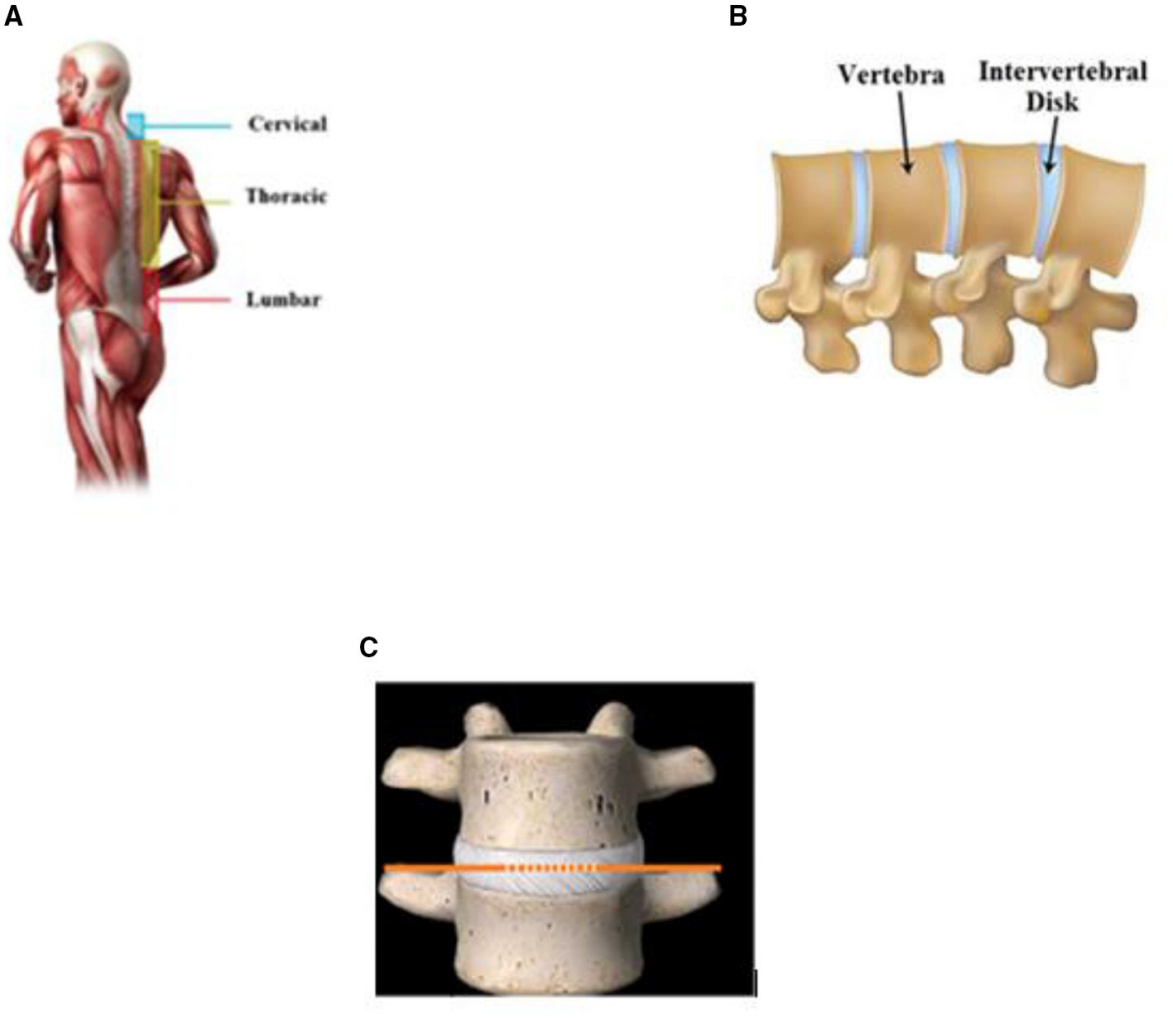
Spinal cord parts of human with integrated FBG sensor **(A)** (57). Intervertebral disk in the spinal cord **(B)** (52). FBG sensor integrated with intervertebral disk **(C)** (57).

The degenerated human intervertebral discs exhibit more compressive stress, tensile stress, and strains. Load distribution on the spinal disc is important for the proper functioning of the lumbar motion of the human body. In this context, Newwell et al. evaluated the use of the FBG sensor in human spinal cord mechanics. Understanding the pressure distribution of the intervertebral disc is a major key point in the clinical diagnostic of disc strain. The biomechanical structure of the spine consists of the intervertebral disc, as shown in Figures 9A–C, between superior and posterior parts of the spine consist of an intervertebral disc. In the study of Newwell et al., biomechanical action took place between these parts. Finite element mandrel was developed with encapsulation of FBG sensor within it. FBG sensor was incorporated with the intervertebral disc part. Compressive or tensile force applied on the intervertebral disc has generated the strain. Encapsulated FBG sensors had undergone pressure that brought the change in the grating period. Functional units of the spinal cord experienced compressive load. FBG sensor incorporated in intervertebral disc and linearity, sensitivity, and hysteresis for each compressive stress was investigated. The porcine intervertebral disc (IVD) pressure has been validated with an FBG sensor for accuracy. Needle mounted strain gauge sensor was inserted into the nucleus. Even though the invasive measurement method was divisive, this method could undergo accurate pressure measurement in the IVD. Functional units of the spine were compressed for 0–500 N force. The substantial potential has been exhibited by FBG sensor with IVD pressure monitoring by sidestepping the controversial nature of invasive methods (58, 59). In another work, Yamaji et al. evaluated the device for analysing the motion of the lumbar. The device consisted of stretchable strain sensors. It was observed from the clinical survey that lower back pain was common in most people in the world. In this study, the stretchable device for lower back motion was analysed. Six strain sensors were used in the human spinal cord in the non-invasive method. Sitting postures were the major cause of most spinal cord diseases. In such cases, detection of the wrong posture could give major advantages. Many wearable devices had been investigated for different applications, wherein the sensitivity of the sensor achieved as high as 0.2 nm/με^−1^ (60, 61).

#### Monitoring Angular Motion of Joint and Gait Analysis Application

Diagnosing issues causing pain during walking or implementing or evaluating pain for treatments is paramount in physiotherapy treatments. Monitoring the angular motion of the joint helps in restoring correct patterns and abnormalities. In this context, Padma et al. proposed a goniometer device with FBG encapsulated for dynamic angular motion of joint. Rotational motion of join in the affected human body was converted into strain variation. Developed FBG sensor was able to detect the range of angular motion from 0 to 200°. The resolution obtained was in the range of 0.06°. Elbow joint and ankle joint measurements were carried during the experimentation. The sensor device was used to measure the angle between the shin and thigh for various cantilever strain variations. Knee angle measurement device was also validated for a range of motion of joint angle in gait analysis with traditional sensors. Sensitivity switching mode was made available to obtain various levels of sensitivity action mode. Low sensitivity mode, medium sensitivity mode, and high sensitivity mode were developed in the device. They also used polymer optical fibre for joint angle measurement. The obtained results were compared with a resistive goniometer and polymer optical fibre sensor (57, 62). In another work, an electro goniometer was used to measure the joint angle. The goniometer was embedded with an FBG sensor to get the angular motion details. However, the drawback of this device was the fixed hinge and fixed centre of rotation which caused a problem during the measurement of angle in joint with more bend. Alternative fibre optic-based device with the technique of intensity modulation and laser beam methodology was developed. The prototype developed had a range of angular rotation up to 90° with a standard deviation of 0.1°. The developed sensor has high reliability, sensitivity, speed, and flexibility (63). In this context, JR Galvao discussed gait analysis with FBG sensor. Gait analysis is the study of the motion of a human or animal body using the eyes of the observer through various study devices for the measurement of motion with body mechanics. Sport and medicine are the widely used area of gait analysis. The walking process, steps in walking, body posture, and walking velocity are the different parameters depending on the gait analysis result. Different gaits of horses had been studied using the instrumentation of the FBG sensor. Early injury diagnosis of horses and retirement of horses were investigated in the said study. Initially, the FBG sensor was used without any encapsulation and embedded in each limb of the horse. In the second step, FBG was encapsulated with composite fibre and embedded in the limbs. The second step involved digital image processing techniques to get proper images of compressive or tensile forces. Both FBG sensing and digital image processing had shown good agreement for biomechanical study and evaluation of FBG sensors for horse dynamic training and competitions (64, 65). Furthermore, the study of Domingues et al. discussed an insole FBG-based optical fibre pressure sensor. Internet of Things technology was incorporated with this application. An optical fibre-based foot pressure system was integrated with the Internet of things for e-health assessment. Accuracy of the system was varied with normal walking and patients with diabetes. Polymer optical fibre was embedded in the foot plantar surface. An array of polymer optical fibre has placed foot insole and experimented for different gait cycles. Four different FBG sensors characterised by four different wavelengths had been used. The FBG sensor was positioned at different positions of insole, i.e., first, second, third, fourth, and fifth metatarsus. Finite element analysis (FEA) was carried out for the insole model. The high sensitivity of 0.000492 nm/KPa was obtained for the developed system. The insole was embedded with multiplexed FBG-based sensor. The designed system was allowing complete gait motion assessment with a multiplexed FBG sensor network. Assessment results of the insole would be sent to a physiotherapist to monitor the conditions of the patient (66–69).

#### Respiratory Pressure Monitoring

Monitoring of respiratory system reduces the severe illness during consideration of clinical response. Exploring the possibilities of good respiratory monitoring sensors such as the FBG system is vital in the present scenarios of chronic respiratory diseases. In this context, Padma et al. discussed a breath pattern analyzer based on the FBG technology for communication assistance for people having restricted communication ability. In emergency medicine for people with spinal cord or brain injuries, or patients in the intensive care unit, critical life-supporting system is required. The FBG breath pattern analyzer was used for the patient to communicate with the outside world, where specific breath patterns were trained by FBG breath pattern analyzer. During emergency assistance, the patient could be communicated for specific purposes. The developed device was an alternative to the brain-computer interface, where specific instruments were used to obtain the brain responses (70). In this context, Krehel developed a textile-based respiratory system. In this system, highly flexible polymeric textile fibre was embedded with an optical fibre pressure sensor, and the pressure applied on optical fibre has shown a distinct shift in wavelength. The device was analysed with different setups with an optical fibre pressure sensor. Half oval with a length of 60, 180, and 240 mm, as well as half oval with additional bends and with additional bend and cross fibre is cosnidered in the design. The pressure was monitored with a human torso. This comparison with different setups was made to assess the utility of this monitoring device (71). In another work, Nishiyama et al. discussed and evaluated respiration and body motion during sleep with FBG technology. This investigation is essential to understand the human breathing pattern during sleep and detect the motion of the body, sleep habits, etc. The optical fibre pressure measurement system was embedded in a conventional bed. Pressure changing due to breath and roll over in bed was monitored throughout the process using plastic optical fibre-based respiratory monitoring system. Plastic optical fibre was used as a sensor to measure the rate of breathing. This optical fibre pressure sensor has been placed in tape form in the chest of the patient to monitor respiratory movements. The input and output signal from the optical fibre pressure sensor was obtained as intensity modulation. The pressure sensor was tested in a chest in normal status, sitting, and lying condition. The proposed sensor has provided different possibilities in respiratory monitoring within the MRI system (72, 73). In this context, Massaroni et al. discussed the method of measuring respiratory rate by the contact-based method. Different respiratory rates were recorded with the help of sensing element. The complete details discussed were about the contact-based methods, monitoring airflow pressure, air humidity, air movement, chest wall movement, and sound. Differential flow metre, turbine flow metre, anemometer, and fibre optic sensor were used during the process. MEMS-based pressure sensor for simultaneous monitoring of breath pressure and pulse wave was analysed. The real-time experiment was conducted on nasal pressure to monitor the pressure developed at the nasal airways. The MEMS-based pressure sensor pad was attached to the human nose. Eyeglasses was attached the sensor pad to the nasal part (74, 75). In another work, polymer optical fibre-based respiratory rate monitoring sensor has been analysed. In this work, a stretchable strain sensor was made wearable around the stomach. During respiration, inhale and exhale process, the stomach would expand. The sensor would measure the strain generated by expansion. Infrared light was passed through the stretchable grating fibre (76).

#### Photonic MEMS Microfluidic System

**Figure 10:**
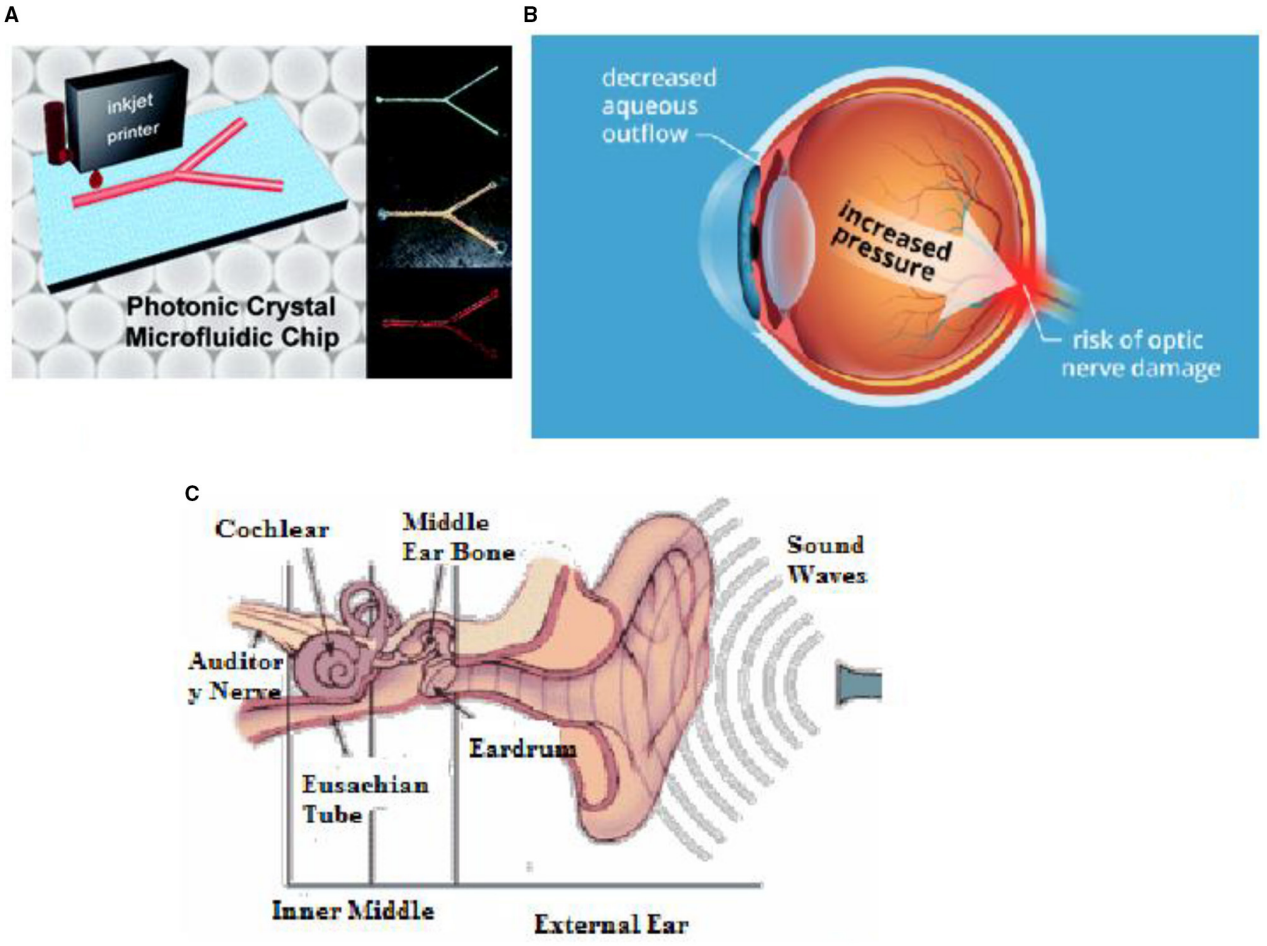
**(A)** Microfluidic channel with photonic crystal integration (81). **(B)** Intraocular pressure development in the eye (82). **(C)** Human cochlear system (83).

Optical MEMS with microfluidic concepts involves techniques of lab on chip-based sensors. The system consists of optics and fluidic systems with mechanical actuation. Microfluidic channels are a vital component of photonic MEMS for any biosensing applications to introduce any type of fluid for sensing. In this context, Jeiang et al. discussed an FBG-based Fabry-Perot pressure sensor in a lab on a chip that included microfluidic channels. As the different polystyrene microparticle size varies, the wavelength shift was observed. Sensor systems presented ease of fabrication methods and label-free detection methods. The sensor was demonstrated in polymethyl siloxane chips. It was also monitored that experiments and analytical results fitted very well within the context and avoided the waste of samples (77). In another work, the FBG sensor was integrated into the microfluidic channel. It provided opportunities for dynamic measurement of the physical and chemical properties of fluid passing through the channel. In another work, Weizhi et al. discussed a photonic crystal-based microfluidic channel fabricated with the inkjet printing process. Photonic crystal with the different coloured colloidal microfluidic channel was successfully integrated into a single chip. The fabricated microfluidic channel has the advantage of rapid fabrication possibility, and colourimetric was the add-on properties of photonic crystal based microfluidic channel (78, 79). In this context, Nunes et al. discussed silica-based 1D photonic crystal in opting fluidic systems. It comprised of photonic crystal with the integration of a microfluidic channel. By considering the nanopillar configuration of the photonic crystal, biofluid was injected into the microfluidic channel during the analysis. In the stage of the simulation, the refractive index was taken into account. The shift in resonant wavelength has been observed during the propagation of light due to a change in the overall refractive index profile of the sensing structure with the microfluidic channel. The author has proposed the sensor for biochemical applications (80). Figure 10A shows the integration of microfluidic channels with photonics. In another work, Khoshnoud et al. discussed the use of MEMS-based biomedical sensors.

Micro-electromechanical system devices are used to detect triglycerides, glucose, and are used for the measurement of pressure during surgeries, counting the blood cells, sensing in the thermal fluid, and electromagnetic fields (84, 85). In this context, Quack and Sattar discussed a nanoscale photonic integrated circuit to explore optomechanics. In this work, the authors mainly focused on reducing power consumption due to the movement of mechanical parts within the system. Due to the minor modulation in the effective refractive index, it was possible to get the required consumption of power. The authors also discussed the recent achievement of MEMS enabled silicon photonic systems (86). In another work, an optofluidic pressure sensor integrated with a microfluidic channel was developed. Optofluidic pressure sensor made up of Fabry-Perot resonator system was integrated with a fluid flow wall. The sensor obtained high-quality factors and high sensitivity with a good detection limit. The sensitivity of the sensor was 12.46 nm/MPa with a detection limit of 8.2 bar. A silicon photonic mid-infrared system with microfluidic channels was integrated. In this system, silicon on the insulator layer was developed. The author focused on increasing sensitivity by increasing the length of the interaction of light with structure, intensity, and reducing noise (87, 88).

#### Measuring Intraocular Pressure

Intraocular pressure (IOP) monitoring is an essential aspect of the human eye to avoid the risk of glaucoma and to provide clear vision to a person. Pressure build-up within the eyes optic nerves is the leading cause of glaucoma. Glaucoma damages the optic nerve, and if untreated, may lead to vision loss, which then makes a person blind. In this context, Lee et al. developed a micro-sized optical implant for *in vivo* intraocular pressure monitoring. Sensors were mounted in intraocular lenses for continuous monitoring of pressure. Sensors were tested with New Zealand white rabbits, and in this way, continuous intraocular pressure measurement was made possible. In another work, intraocular pressure sensor was used with the technology of a flexible photonic crystal membrane.

Periodically, micro-structured 1D photonic crystal on polydimethylsiloxane was considered in the experimentation process. The estimated limit of detection was about 160 Pa. The micro-size compatibility of these sensors has been rendered as a favourable application for glaucoma. Figure 10B shows intraocular pressure development. IOP sensor was integrated into keratoprostheses, and the proposed sensor has given 2 mm/Hg accuracy (89–92). In the study of Han et al., they discussed an optical aberrations effect on intraocular pressure measurement with small optical elements in this context. Elevated intraocular pressure is a significant risk factor for glaucoma. To monitor this, the implantable optical pressure sensor was incorporated *in vivo* with rabbit eyes. Optical aberrations relationship with intraocular pressure sensor performance was monitored. It was concluded that sensor readout accuracy decreases as the magnitude of optical aberrations increases. The accuracy of signal to noise ratio obtained was 0.58 ± 0.32 mmHg and 15.57 ± 4.85 dB^3^. In another work, Truong et al. developed a portable handheld reader for monitoring intraocular pressure. Reflected light from the intraocular pressure sensor forms fringed and it was captured using a camera. Three different handheld readers were used to analyze the better quality fringes and patterns. It was concluded from the results that DSLR based reader has high-quality fringes compared with others. Monitoring of intraocular pressure with optical coherence tomography is a novel technique. Hypertension can lead to glaucoma. Frequent control of IOP monitoring corneal deflection either by direct contact or non-contact process is the current procedure involved in clinical treatment. The relationship between OCT images and the IOP sensor was developed by capturing the bovine eye (81, 93).

#### Blood Pressure Measurement

Daily monitoring pulse rate is essential to success to meet fit and healthy lifestyles, especially for people with cardiovascular diseases or weight loss. FBG based system is widely applicable for monitoring pulse signals because of its micro size and high sensitivity to external pressure. The study of Katsuragawa et al. evaluated FBG based non-invasive blood pressure sensor. Systolic pressure and diastolic pressure pulse were measured in a single FBG pressure sensor. FBG sensor was kept at the right wrist and right elbow. Results were validated regarding blood pressure values. The device developed had good agreement with standards of sphygmomanometer. In this context, Opsens' OPP-M developed a cardiovascular blood pressure monitoring system using a fibre optic sensor. Dynamic temperature measurement was also made possible with this device, and less moisture was induced during the monitoring process. A reduction in size makes the device more favourable for invasive cases. Devices possessed immune to electromagnetic interference, magnetic resonance imaging, and any other invasive surgery instruments. The integration of the sensor was made easy with a cost-effective strategy. The optical fibre pressure sensor used an optical detector, light source, signal processing module, etc. It was observed that the blood pressure measurement based fibre optic system is reliable and faster in terms of sensitivity and quality factors. Most promising technologies and improvements carried under optical MEMS systems for blood pressure measurement applications used the concept of FBG system, fibre optic system, optical ring resonator, photonic crystal membrane, and interferometers (94, 95). Silica optical fibre-based FBG sensor was designed and discussed for strain measurement. Induced pressure by pulse waves from human arteries is the signal for the FBG sensor. Since silica optical fibre may undergo fatigue fractures, plastic optical fibre was introduced to resolve this issue. Pulse waveform from the human arteries was obtained. Based on the results obtained, the measurement of waveform plastic optical FBG was the better solution compared with others in terms of accuracy and sensitivity (96)^4^. In another work, Katayama et al. proposed wearable blood pressure measurement for tracing the sudden change in human blood pressure in the arteries. FBG optical pressure sensor was embedded in the textile or garments for the easy measure of pressure waves form in the human body. The partial least square method was followed for calibrations of pulse waveform and induced FBG pressure. The sensing accuracy obtained by this sensor was comparable to conventional sensors (82, 96, 97)^4^.

#### Bladder Pressure Monitoring

We know that pressure is a critical parameter that is used to represent the state of all organs in the body. Hence the measure of pressure is very crucial in the medical field. The conventional system uses the fluid base wherein the pressure is indirectly transmitted from the required organ. These often have a delay in measurement since fluid lines are used as a medium of measurement. The traditional methods of measuring pressure have always proven to have minor errors. Usually, there are no sensors places in the body of a patient since it limits the movement of a patient. Hence, pressure is measured when the person is hospitalised or before and after a problem. Due to these factors, we do not have any constant measuring of pressure in the human body, and it does not reveal the underlying problems faced by people. In this context, Poeggel discussed the urodynamic analysis for evaluating the dysfunction of the lower urinary tract during the filling and voiding process of the bladder. Pressure measurement of the bladder during this process made the urologist provide a required treatment to a patient based on individual diseases. The technique was based on two optical pressure sensors and a temperature sensor. A real-time pressure measurement system was made possible by this method. To overcome this difficulty, we can design lightweight, small-sized, and efficient MEMS. MEMS can be used with minimal efforts using invasive techniques that can continuously monitor the condition of the patient all throughout. Urinary incontinence, which is the involuntary loss of urine from the bladder, occurs in ~10% of the male population worldwide. This procedure was performed by a skilled professional. The procedure consisted of inserting a catheter into the urethra of a patient into the urinary bladder, where there is a transducer in one end. The pressure and the reference pressure were measured in the rectum through fluid lines. The target pressure of the urethra was compared with the reference pressure of the rectum and analysed. It has a substantial margin of error due to the movement and measurement errors, however, pressure is a vital parameter to measure the performance of organs. For effective diagnostic, high quality and high resolution in measurements is necessary. In organs such as the urinary bladder, water-filled lines have their limitations and have still been practised throughout the decades. We should make use of the advancement in photonic MEMS, which offers a very small pressure sensor with precision and accuracy. We could conclude that there are more limitations to the values measured with water-filled lines. Hence, an in-target pressure sensor from the organs is much advanced and reliable in detecting any changes. In the meantime, optical fibre measurement systems are also considered for monitoring the temperature. The system consists of an encapsulated fibre optic pressure sensor and a Fabry-Perot pressure sensor. The optical fibre used in this case is FBG pressure sensors. The optical fibre pressure measurement system is compatible with the clinical phase. The whole system is compared with the traditional biocompatible urodynamic measuring system (98).

#### Cochlear Implant Application

Deafness affects about 10% of the general population, and the rate increases to more than 30% for those aged over 65. However, a hearing prosthesis such as a cochlear implant can provide shearing and significantly improve the quality of life. It is the most important advancement in the treatment of hearing impairment. In another work, Yao developed a fibre optic-based bending sensor for cochlear implantation. It has proved a great success in providing implantation for deafness in children and adults. Implantation using a cochlear electrode array is a complex surgical process, and there is a chance of damage to the inner wall of the cochlear, i.e., scala tympani. Since the electrode has to bend at various angles, and if the electrode does not bend at the required angle, damage to the cochlear may be severe. To avoid this, researchers developed a bend sensor for implantation surgery (99). In this context, Balster has investigated hearing restoration by optical stimulation alternative to electric stimulation. Activation of the auditory system was made by light fibre insertion into the coiled cochlea. Light fibre force measurement was conducted, and results were compared with traditional electric stimulation study. The results showed that minimal painful implantation surgery optical stimulation was considered feasible (100). In another work, fibre optic-based intracochlear pressure measurement system was developed. A 81 μm-diameter superluminescent diode (SLED) and other modes of optical fibre were embedded inside the transducers. These cochlear fibre optic sensors were helpful for early monitoring of hearing loss, as well as the sound pressure measurement of temporal bones and mechanical stimulation of the intracochlear system (83, 101, 102).

The work of Starovoyt et al. discussed optical coherence tomography for intracochlear structure. Studying the temporal bone by using OCT was the main aim of the authors. The authors focused on obtaining high-resolution images using optical coherence tomography qualitative and quantitative characteristics of intracochlear structures. The importance of OCT images in intracochlear implant surgery was explored. Implantable sensors for hearing devices and wearing the external hearing aid causes discomfort for most people. Different designs of the implantable sensor were evaluated and compared (103–105). Figure 10C shows the view of the human cochlear system. Key applications of optical MEMS sensors are presented in Table 2.

**Table 2:** Key applications of Optical MEMS in biomedical.

**Medical area**	**Type of modulation**	**Place**	**Monitoring strategy**	**Technical feature of sensor**	**References**
Dental	Optical Coherence Tomography	*In vitro*	Carries, cracked tooth	B-mode PAT is good at providing microstructural properties. S – Mode PAT provides a proper imaging system of the crack lesion.	(53)
	Fibre Bragg Grating	*In vitro*	Force monitoring in maxilla model	Force range detected 0–20 N	(51)
Intervertebral disc	Fibre Bragg Grating	*In vitro*	Pressure monitoring in intervertebral disc	Pressure range between 0 and 500 N	(59)
Lumbar motion	Fibre Bragg grating	*In vitro*	Lumbar stretch monitoring	Rotation angle upto 0–35°C	(60)
Low back motion	Optical fibre	*In vitro*	Low back movements monitoring	Sensitivity 0.20 nm·mε^−1^	(61)
Join angle	Fibre Bragg grating	*In vitro*	Goniometer	Joint angles in the range of 0–200° with a resolution of 0.06°	(65)
Insole pressure	Optical fibre	*In vitro*	Insole force monitoring	Sensitivity 11.06 pm/N	(66)
Communication assistance	Optical fibre	*In vitro*	Using breath pattern analyser	Average accuracy of the device obtained 90%	(67)
Pulse wave and respiratory	MEMS + Optical	*In vitro*	Pulse pressure monitoring	Minimum detectable pressure 0.01 Pa	(74)
Microfluidic	Photonic crystal	*In vitro*	Fluid flow sensing	Sensitivity 836 nm/RIU	(87)
Intraocular	Optical MEMS	*In vitro*	Glaucoma Monitoring	accuracy of 0.29 mm	(90)
Oximetry	Optical Fibre	*In vitro*	Oxygen monitoring by the contact force	Detection limit of 5-15KPa	(96)
Pulse wave monitoring	Optical fibre and FBG	*In vitro*	Pulse wave signals	POF-FBG APG correlation is distributed from 0.54 to 0.72	(82)
Urodynamic measurement	Optical fibre pressure sensor	*In vitro*	Urodynamic Pressure	0.1 cm H_2_O (~10 Pa), a stability better than 1 cm H_2_O/h	(98)

### Other Applications

The study of Samavati et al. discussed the development of the COVID-19 virus diagnosis kit using a novel Go- decorated Au/FBG sensor. The saliva of a patient is considered a favourable testing specimen for COVID-19. In this study, salivary samples was collected from different types of people. The FBG-based probe has been used to detect the COVID-19 virus. The FBG probe was immersed in healthy saliva and saliva with the COVID-19 virus. The FBG probe detected the presence of the virus. The density of the virus was 1.2 × 10^8^ copies/ml in saliva. The maximum change in wavelength observed was 1.12 nm and an intensity change of 2.01 dB (106). In this context, Sharath U designed and developed different types of FBG devices for novel medical applications. The author designed an FBG pulse recorder for cardiology applications and individual bite force monitoring devices. Bite force in men and women has been differentiated with the help of this device (107). By considering the same concept, author Jha et al. proposed an FBG sensor-based glove to monitor the flexure of 10 finger joints. The author evaluated the accuracy and repeatability of all 10 sensors. The FBG-based glove has been designed and tested with an FBG interrogator. Stroke patients were benefitted from the device developed (108). Similarly, in another work carried by Alwin T and co-authors, FBG sensor was used as a multi ocular sensor. In this case, FBG was utilised for sensing temperature, pressure, and chemical concentration (109). The human respiration rate was monitored with FBG based sensor with an Arduino interface. The respiration rate was also calculated during coughing and handshaking. Compared with an electric sensor, FBG-based sensor has shown prominent results (110). In this context Tavares et al. embedded an FBG sensor in a wheelchair. This wheelchair was mainly used to monitor the ulcer in bone areas of the seat. The FBG sensor was also used for sensing dorsal flexion and extension movements. Spending the time in job for many hours with bad posture will lead to back musculoskeletal disorders. Thus, FBG sensing that was developed has shown promising results for future fabrication (111, 112).

In another work, the authors developed a system for monitoring soft finger deformation, which was driven by shape memory alloy with an FBG sensor embedded. Firstly the structural and mechanical properties of a finger were considered for analysis. The central wavelength for change in curvature of material due to shape memory alloy deformation was monitored with FBG sensors. Soft finger deformation was analysed with ABAQUS software for analysing the FEA simulations (113). In this context, Mo et al. designed optical sensing needles for tissue identification. Optical sensing needles were used for minimally invasive surgery. Optical sensors were mainly used because of their immune to magnetic resonance. Intensity-modulated Fabry-Perot interference was considered for optical needles. Developed optical sensing needles were successfully implemented in the medical field (114). Application of intravascular catheterization was used in minimal vascular surgery considered distal force sensing system. The designed sensor has shown higher repeatability and stability. With the distal tip force sensor, it was obtained from the result that the stability of each flexure movement could be controlled with the FBG-based sensor (115). Non-invasive methods to measure wrist angular handgrip devices had been developed to measure the handgrip strength of humans with muscular-skeletal disorders. Handgrip strength was measured in different angular positions. The FBG sensor was encapsulated in the mechanical package and its variation in strain with peak resonance wavelength was monitored for various ranges of applied pressure (116).

**Figure 11:**
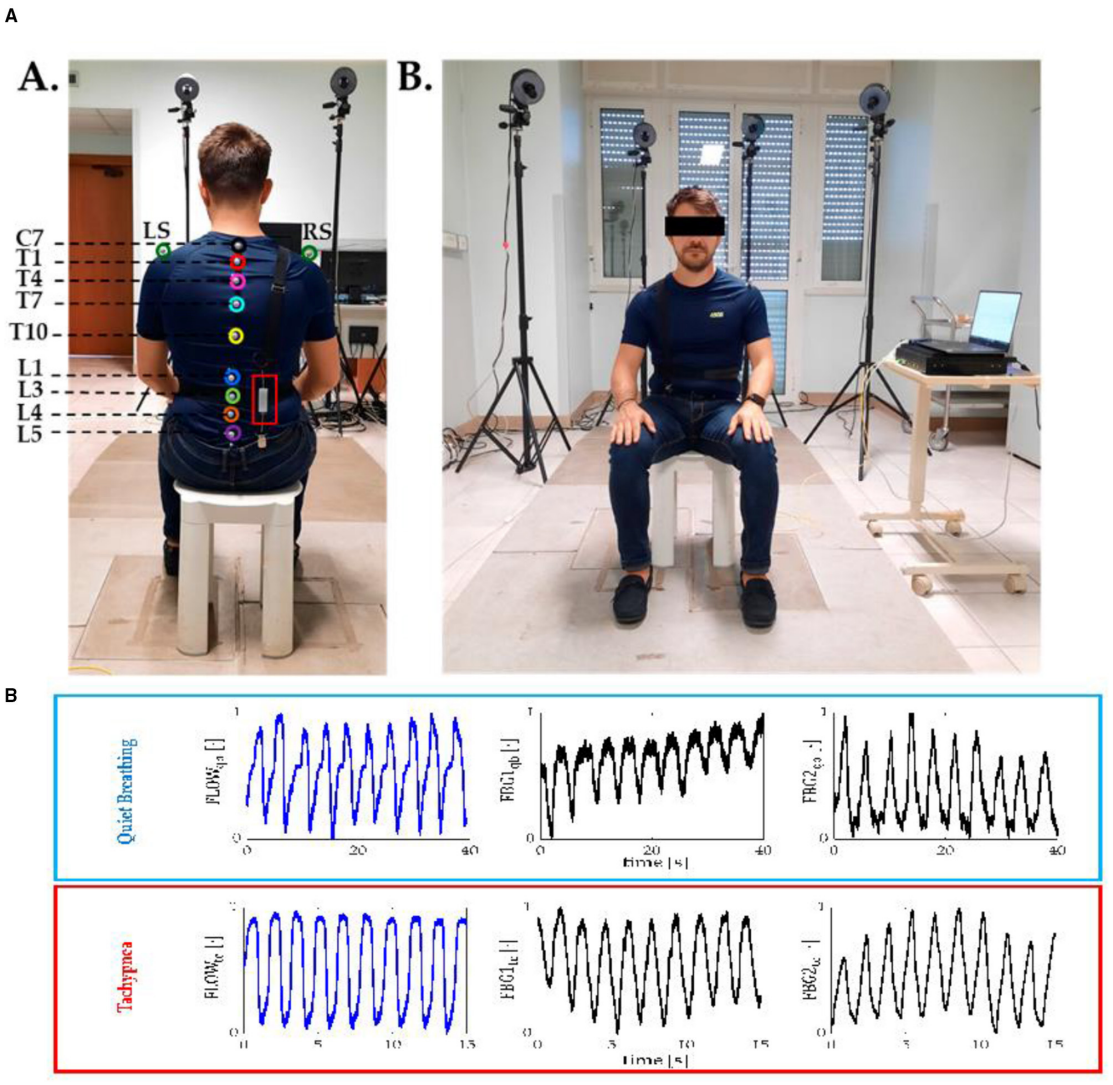
**(A)** Back view showing the posterior part of the wearable devices with embedded sensor and and front view showing the anterior part of the embedded sensor (118). **(B)** Signal collected by flow meter and FBG during quite breathing and tachypnea (119).

Lower back pain is one of the most common muscular-skeletal disorders affecting workers. Video terminal workers are the most affected people with lower back pain. Prompt detection of wrong seating postures is more critical. A lumbar movement monitoring device has been developed with FBG sensing technology. The sensitivity of the FBG device developed was 0.20 nm/mε^−1^. This is the first device with silica embedded FBG sensing technology for lumbar motion monitoring for worker safety to overcome lower back pain. Figure 11 shows the wearable device installed in person back for experimental purposes. The study of Lo Presti et al. considered an FBG sesor embedded in silica gel for monitoring neck movements and respiratory frequency. Neck movements during flexion-extension were investigated. In the meantime, FBG sensors were embedded in the human chest and monitored during quiet breathing and tachypnea (61, 117). Figure 11A shows the different views of a person with a wearable device for experimentation.

**Figure 12:**
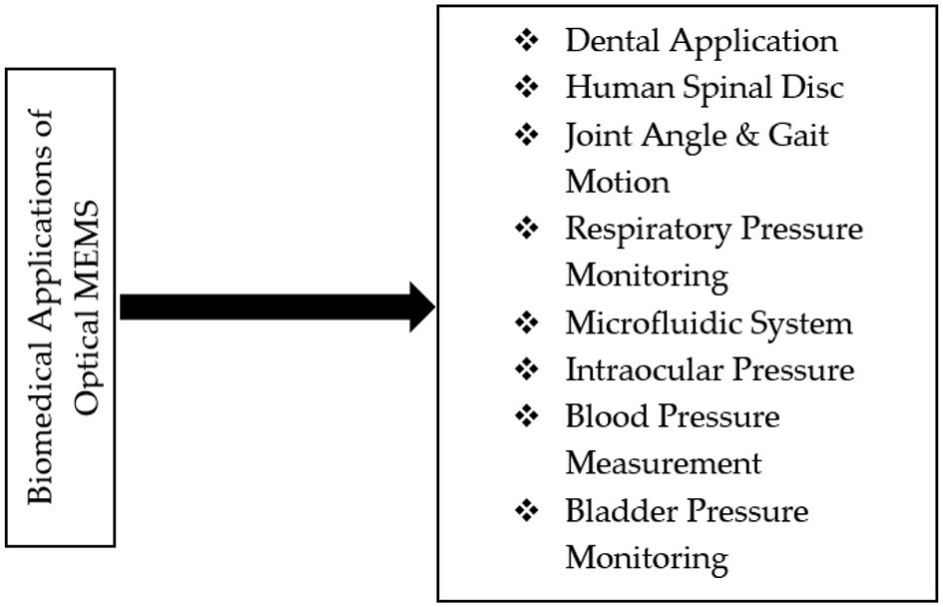
Major applications of optical MEMS in biomedical.

In the same context, FBG sensors are used in human gait analysis, including diagnosis, rehabilitation, and sports. The FBG sensor encapsulated in the mechanical package was used to monitor the extension and flexion movement. This evaluation is useful in monitoring walking disorders and pathologies. FBG wearable system was developed with flexible polymer matrix is embedded with FBG arrays as knee embrace. The developed FBG wearable system has shown sensitivity up to 3.944 nm-mm^−1^ (120). Figure 11B shows the signal collected by the flow metre and FBG during experimentation. In another work, Ran et al. proposed an FBG-based immunosensor for cardiac biomarkers. The concept of temperature cross-sensitivity was considered during the development of proof of concept. The tool was used to identifying acute heart diseases. In a similar case, FBG sensors were embedded in T-shirts to monitor the respiratory rate during cycling. Massaroni et al. proposed an FBG sensing system embedded in cloth for monitoring human body breath monitoring. Twelve FBG based sensors were embedded in the T-shirts. Four volunteers were considered during the study (49, 118, 119, 121–125)^5^. FBG sensors are also used to measure the pose of different medical instruments used in surgery, wherein FBG sensors are embedded in catheters. Flexible movement of catheters during surgery is monitored with an FBG sensing system (126–128). Table 3 shows the summary of the application. Figure 12 shows the major application of optical MEMS.

**Table 3:** Summary of application.

**Sl. No**	**Application**	**Summary of results**
1	Dental	❖ Prediction of dental carries using optical sensor ❖ Incorporation of FBF sensor for dental carries ❖ Strain pattern evaluates for different parts of dental region ❖ Evaluation of dental filler material with FBG ❖ Evaluation of biting force with FBG sensor
2	Intervertebral disc	❖ Load distribution over intertribal disc during tensile and compressive stress has been monitored. ❖ Incorporation of FBG sensor spinal cord in different sitting position
3	Lumbar motion	❖ Load distribution over lumbar disc during tensile and compressive stress has been monitored. ❖ Incorporation of FBG sensor in lumbar position
4	Joint angle, gait analysis, insole pressure	❖ Integration of FBG sensor in insole for foot pressure monitoring in diabetic and arthritis patients. ❖ Embedding the sensors in insole and checking pressure distribution for different types of foot and in different experimental conditions such as walking, standing, climbing, age, and sex and in different patients.
5	Communication assistance	❖ Development of communication assistance based on eye blinking integrated with FBG sensor signals
6	Pulse wave and respiratory, body pressure	❖ Different pressure points of the body are monitored with FBG sensor ❖ Respiratory pressure is monitored with FBG sensor using wrapping the FBG sensor based belt around chest
7	Intraocular	❖ Optical fibre based sensor has been used to detect the glaucoma in patients
8	Urodynamic measurement	❖ Optical fibre has been incorporated with catheter to
9	Microfluidic system	❖ FBG sensor has been integrated with different microfluidic channel for biosensing applications
10	Handgrip	❖ FBG sensor for integrated with gloves, designed handgrip models,

## Key Impacts and the Challenges of the Optical MEMS for the Biomedical Application

**Figure 13:**
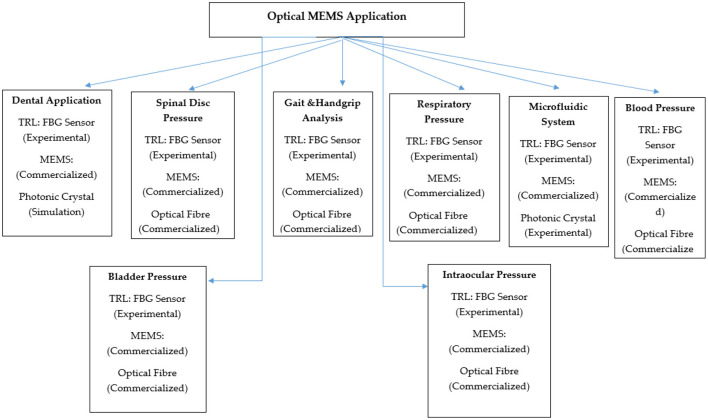
Major applications and TRL (technology readiness level) of optical MEMS sensing system in biomedical.

The authors investigated crucial factors of the optical MEMS sensing system by considering many research papers and concluded three key elements. These key factors are based on optical MEMS devices, optical MEMS applications, and current optical MEMS technology trends. A detailed analysis of these critical factors is grouped in Table 4. Conventional MEMS sensor uses piezoelectric or shapes memory alloy, transducers to convert physical phenomena and into electrical signals; these signals are scaled, conditioned, and digitised to the required applications. Despite their advantages, these sensors are impractical to use in all applications. Instead, the optical MEMS sensor offers excellent solutions to many challenges faced by MEMS sensors. Sometimes, the optical MEMS sensor works similarly to the MEMS sensor but uses light instead of electricity and silica glass instead of copper. It was observed from the literature that most biomedical applications use optical fibre or FBG technologies for monitoring pressure, temperature, and many other physical parameters. Different optical MEMS systems, such as photonic integrated microcantilevers, MZI, and ring resonators, can penetrate as emerging technology until laboratory testing costs and complexity and reliability issues of these sensing systems are resolved. Critical success factors of the optical MEMS till 2020 include evaluation of sensor performance such as its sensitivity, Q factor, detection limit, Optimization of various sensing structures, etc. Table 4 shows that research work carried out extensively with optical sensing systems such as photonic crystal structures, MZIs, and optical microcantilevers. Performance enhancement of various optical MEMS sensing structures has been carried out. It was possible to successfully implement the FBG and an optical fibre sensing system in many real-time applications. Still, authors are provided scope for improvement in much research work. Feasible sensitivity and detection limit is achieved based on a specific application. Most of the sensors were designed and analysed with transverse electric mode. It is noted that real-time implementation of other sensing systems apart from FBG and optical fibre sensor requires plenty of sensing system characterisation. Figure 13 shows major applications of optical MEMS in biomedical.

**Table 4:** Critical factors of optical MEMS.

**Key factors for the optical MEMS methods/designs**	**References**	**Total studies**
Key factor 1	(1–49)	49 papers
Key factor 2	(50–104, 129)^1^,^2^,^3^,^4^,^5^	60 papers
Key factor 3	(49, 118, 119, 123–128)	28 papers

Three critical factors are identified in the survey of optical MEMS for biomedical applications, and are clearly distinguished as Key factors 1, 2, and 3. Research articles on Key factor 1 highlighted the issue of pre-fabrication stages, such as design and simulations. Performance evaluation of sensors in terms of sensitivity, detection limit by design optimization, modified refractive index, and different material assignment for the sensing structure is discussed in Key factor 1. Applications in which optical MEMS sensors have been successfully implemented is considered as Key factor 2. From Key factor 2, it is observed that there is substantial usage of optical fibre sensors, FBG, and MEMS-based optical coherence tomography, optical MEMS actuators, etc. survey on the present market status of optical MEMS are discussed in Key factor 3. The optical MEMS design and testing phase faces technical challenges in the presence of mechanical elements during the integration process involving flow, pressure, movement, and analysing temperature. The combination of the multi-function device makes the design and testing complex during the development process of the optical MEMS sensor. Most organisations have no access to the fabrication facility, even if there is interest in exploring the technologies. Packaging can be expensive and knowledge of fabrication challenging aspect in the case of optical MEMS.

**Figure 14:**
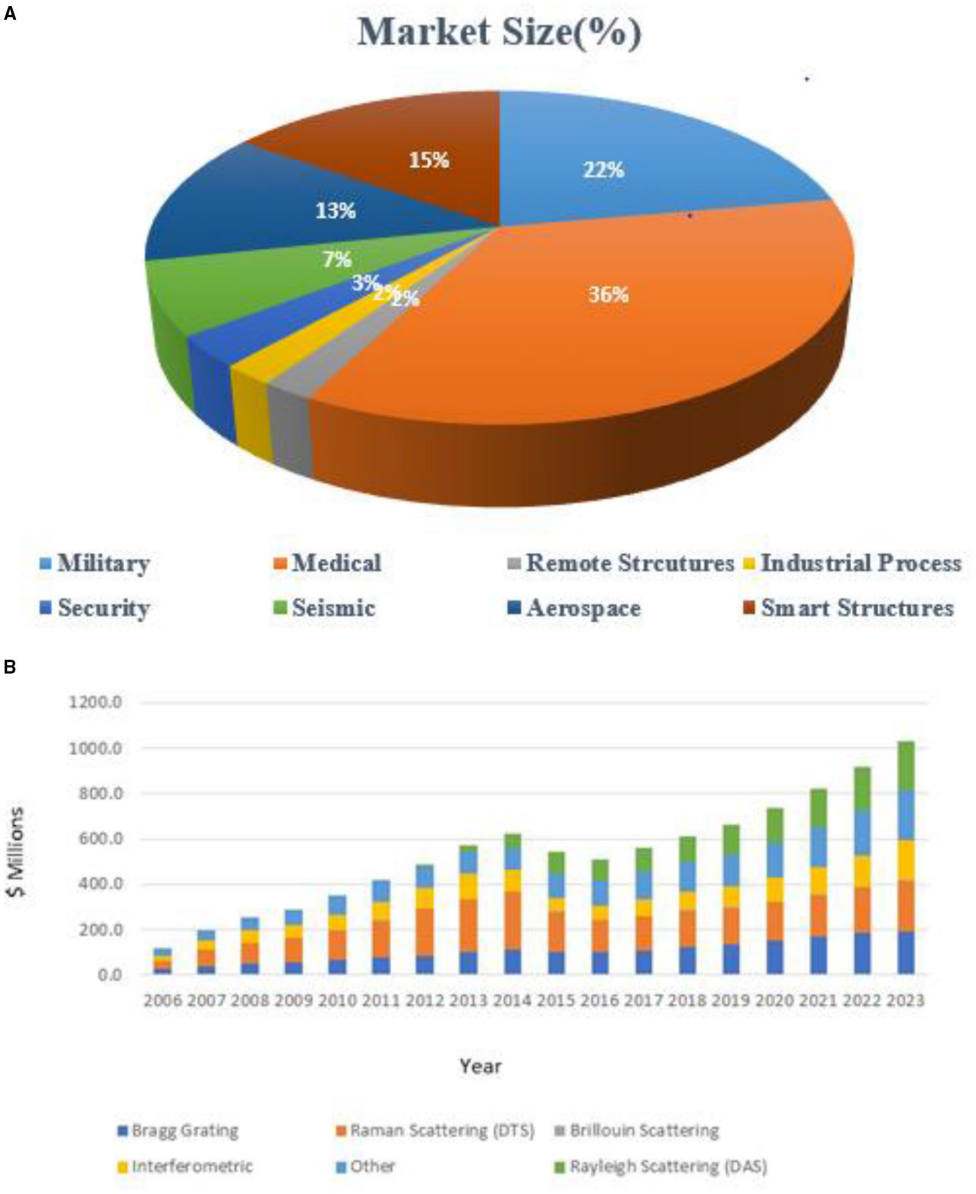
**(A)** Presents trend of Optical MEMS market (127). **(B)** Optical MEMS market growth until 2023 (127–133).

An overview of optical MEMS market growth in 2019 offers a complete analysis for better understanding the completion in the market and implementing the same in research activities. The optical MEMS market fragmented in different major companies like S.T. Microelectronics, Analogue Devices, Freescale Semiconductor, Texas Instruments, Boston Micromachines, and Memscap. As shown in Figures 14A,B below, the maximum market size of optical MEMS in the current era in the field of Biomedical Engineering is preceded by Defence and Security and Smart structures. The least volume of the market is observed in the small structures. Under the optical MEMS category, the original product is the projection system, microbolometers, MZI, optical cantilevers, ring resonators, and FBG sensors.

The fibre optic sensor market is projected in 2017 to be $1,008 million in 2021 and reaching above $1,033 million in 2023. Most commercial efforts focused on medical and military applications. Due to the continuous improvement in the FBG writing system, there is an increase in demand. Among the different types of FBG types, uniform FBG type is expected to have compound annual growth rate over 14% as forecast in upcoming days. Photonic crystal biosensor, optical ring resonators type of sensing system has shown less trend presently due to sophisticated experimental setup and more importantly because of the technology readiness level of photonic sensing devices. Most of the photonic crystal sensor has to be cascaded which leads to the difficulty over fabrication. Bringing out the photonic sensing system in the main market, except for fibre optics-based sensors, takes few years of time due to this challenging process involved. The global market of photonic crystals has reached up to $43.119 billion during 2015 and it will be $60.230 million during 2022. Photonic crystal sensing research has got attention from other academicians, as well as from scientists. Research filed such as industrial telecommunications, biomedical field, LED display, and optical sensing technology has shown remarkable growth with photonic crystal from the past few years.

Obstacles for the growth of the optical MEMS device mainly include design complexity, reliability, fabrication access, packaging facility, and knowledge of fabrication. The optical output parameters such as Q-factor, sensitivity, and transmission efficiency mainly depend upon the final packaging of the device. Since the optical MEMS technology involves more moving parts controlling the device, accuracy requires expertise in a multidisciplinary field. Despite these challenges in optical MEMS technology, it overcomes many disadvantages faced by MEMS technology, such as high sensitivity, less power consumption, EMI Insensitivity, and lightwave manipulation can be more comfortable with moving mechanical elements. The device-level design, fabrication, or packaging complexity is due to less exposure to optical MEMS technologies. Simplifying the device design and packaging process is the scope in the optical MEMS technologies. Figures 14A,B shows the curret trends of optic MEMS sensor in market.

Simplifying moving micromechanical parts in terms of size is essential for increasing the efficiency and performance of optical MEMS. Design and optimization of moving mechanical elements essential criteria to build the integrated optical sensing system. Using the sophisticated fabrication lab software tools for pre-checking the final model and packaging for reliability, the accuracy of readings is the primary solution to speed up the final fabrication to bring the device technology to commercial status. This technique mainly applies to the photonic sensing system in the technology readiness level as experimental such as photonic crystal, photonic ring resonators, and MZI. In the case of FBG based sensing system, miniaturisation of FBG interrogator device in the form of chip will increase the demand in the market. Accurate debugging and design tuning, preparing virtual prototypes, Gathering practical feedback at the time of designing are the factors making up the successful implementation of the product. Designing the photonic integrated circuit with photonic crystals sensing technology and optical resonators or MZI is always challenging; actual implementations are possible with 3D emulators. This process shows how the microchip will work in 3D visualisation and helps the final working of the product after packaging. There will be unpredictable and immeasurable errors that have to be overcome for ease of fabrication. High tolerance needs to be maintained in the design for achieving targeted output. Coupling problem, temperature influence, controlling size, and position of holes are critical parameters affecting sensor performance due to the requirement of nanometer precisions scale.

## Scope of Future Work

The focus of this review was to explore the recent advancement of optical MEMS sensors and their application in the biomedical field. Key and important issues with optical MEMS had been identified. Using high sensitive MOEMS sensor for the harsh environment have operational and stability issues. There is always scope for developing sophisticated MOEMS sensing structures by analytical and simulation models to improvise the sensitivity, stability, and printable or packaging issues. Optomechanical sensors with high sensitivity could be developed by considering different mechanical properties, stiffness, the mass of sensing material, etc., thereby increasing response frequency for sensing pressure and displacement. The operational stability of the MOEMS sensor could also be increased by the coating of the sensing layer with different materials. There is always scope to investigate the integration of optical sensing layers with composite and organic materials to make packaging easier. Even though the FBG sensing system has reached commercialisation, the miniaturisation of the FBG system such as interrogator is the requirement of the present scenario to bring compact devices in the market.

❖ Variety of Photonic MEMS sensing systems have been considered in the literature, but minimal sensing system is implemented in biomedical applications due to sophisticated fabrication feasibility.
❖ There is a lot of scope for optimization of photonic MEMS sensing structure for implementation in biomedical applications since most of the photonic MEMS system, apart from optical fibre, is implemented in telecommunication.
❖ Fibre Bragg Grating is a mature technology and has plenty of scope for incorporating in biomedical applications to increase the accuracy, sensitivity, and durability of medical instruments.
❖ Recently published articles show possible future work in terms of design, different experimental conditions for specific applications.
❖ The obstacles to overcome soon include collecting experimental data, fabrication knowledge, and lack of fabrication facility.
❖ Dynamic analysis of each moving mechanical element is necessary before integrating these elements into the optical system to obtain more accurate results.
❖ Design and packaging, testing, sensing, latching, controllability, and reliability are the main challenges in developing optical mems sensors.
❖ Optical MEMS helps accelerate the deployment of different sensing systems in the biomedical field and other structural health monitoring fields.

## Conclusion

This article reviewed different optical MEMS sensors and their application in the various biomedical fields with future trends and growth. To assist clinicians in knowing about the recent developments under optical MEMS, a clear possible visualisation was provided to implement the same technologies in the biomedical field. This paper has investigated the necessary information about optical MEMS, and its possible applications in the biomedical field have been assembled and discussed in detail. With many research articles under microcantilever-based optical MEMS diaphragm, interferometers under different forms were limited to simulation stages, but still showed possible future improvement towards improvising sensitivity with novel structure with experimental setups. Photonic crystal sensing and optical ring resonators had shown more discussion on developing mathematical modelling and numerical simulations. Structural optimization in terms of material properties, geometrical changes, and possible applications has been considered. Experimental techniques were considered in the field of photonic crystal, ring resonator, and MZI technologies. However, readers who are interested in readings of these photonic technologies would find plenty of unique design properties assigned during the structural optimization. Designers had increased their horizons towards finding new solutions for ease fabrication by simple designs with practical outputs. In the near future, photonic sensing technologies will control, integrate the network, and find real-time measurements in different biosensing applications. From the literature, it was also observed that most biomedical applications discussed here use the non-invasive method of parameter monitoring. There is always a possibility of the improvement of non-invasive clinical diagnosing with the optical sensor. Irrespective of complexity in fabrication, optical MEMS has consistently shown high operation speed, high optical efficiency, optical precision, reliability, readability, and scalability. Significant properties the optical MEMS material should incorporate are high stiffness, high fracture strength, fracture toughness, temperature, and chemical inertness. It was also found that optical coherence tomography major equipment used in biomedical image study. Although researchers had understood optical MEMS and experimented with a different application, implementation of the same in reality has not been possible in many cases due to sophisticated technology, lack of experimental setups, and the requirement of testing its consistency in performance. Since there are various fibre optic FBGs sensors, choosing the appropriate coating material for a suitable application is critical. As per the market survey, it was seen that there will always be an increase in demand for optical MEMS sensors with technical advancement.

## Data Availability Statement

The original contributions presented in the study are included in the article/supplementary material, further inquiries can be directed to the corresponding author/s.

## Author Contributions

AU, MH, MS, PS, and SI: writing, methodology, review, supervision, and project management. MH, RH, SI, SA-K, AS, and NV: review and funding. All authors contributed to the article and approved the submitted version.

## Funding

This research has been collaborated and supported by the Faculty of Information Science and Technology, Universiti Kebangsaan Malaysia under the Grant GGPM 2020 028, Taif University (TURSP- 2020/154), Saudi Arabia, Amity University, India, and the Oxford College of Engineering, India. And also this research was supported by the research grant of The National University of Malaysia (UKM) under the Grant GGPM 2020 028.

## Conflict of Interest

The authors declare that the research was conducted in the absence of any commercial or financial relationships that could be construed as a potential conflict of interest.

## Publisher's Note

All claims expressed in this article are solely those of the authors and do not necessarily represent those of their affiliated organizations, or those of the publisher, the editors and the reviewers. Any product that may be evaluated in this article, or claim that may be made by its manufacturer, is not guaranteed or endorsed by the publisher.

## Footnotes

^1^Istock. Avaialble online at: https://www.istockphoto.com/in (accessed July 20, 2019).

^2^Available online at: https://grabcad.com/ (accessed July 27, 2020).

^3^Available online at: https://www.allaboutvision.com/conditions/hypertension.htm (accessed July 27, 2020).

^4^*Opsen's Solution*. Available online at: https://opsens-solutions.com/ (accessed July 20, 2019).

^5^*Distributed and Single Point Fibre Optic Sensing Systems Forecast*. Available online at: http://www.igigroup.com/ (accessed July 20, 2019).
